# Artificial neural network (ANN) based prediction of proppant settling in horizontal wellbores during hydraulic fracturing

**DOI:** 10.1038/s41598-025-26458-3

**Published:** 2025-11-27

**Authors:** Mahmoud Jumaa, Shabeeb Alajmei, Amjed Hassan, Ashtiwi Bahri

**Affiliations:** 1 Department of Petroleum Engineering, King Fahd University of Petroleum and Minerals, Dhahran, 31261 Saudi Arabia; 2 Center for Integrative Petroleum Research, King Fahd University of Petroleum and Minerals, Dhahran, 31261 Saudi Arabia; 3 Colorado School of Mines, Golden, 80401 USA; 4 Amjed Hassan − Center for Integrative Petroleum Research, King Fahd University of Petroleum and Minerals, Dhahran, 31261 Saudi Arabia; 5 Department of Petroleum Engineering, Colorado School of Mines, Golden, Golden 80401 USA

**Keywords:** Artificial neural networks (ANN), Sand settling, Proppant transport, Horizontal wellbores, Hydraulic fracturing, Energy science and technology, Engineering, Mathematics and computing, Solid Earth sciences

## Abstract

Proppant transport and settling in horizontal wellbores is a major challenge in hydraulic fracturing, leading to problems such as sand production, equipment wear, wellbore blockages, and reduced production rates. Traditional empirical models are often limited in accuracy because the physical relationships involved are highly nonlinear and complex. In this study, Artificial Neural Networks (ANNs) were used to develop predictive models for sand settling in horizontal wellbores during hydraulic fracturing. In this work, the data were obtained from controlled laboratory experiments that simulated horizontal wellbore sections with different perforation clusters. Key parameters such as proppant diameter, injection rate, perforation orientation, and number of perforations were analyzed. Two ANN models were developed: Model A used all nine measured parameters, while Model B used five selected parameters identified through correlation analysis. Model performance was evaluated using statistical metrics including the coefficient of determination (R²), Average Absolute Difference (AAD), Root Mean Squared Error (RMSE), and Mean Absolute Percentage Error (MAPE). Additionally, comparative analyses with Random Forest and Gradient Boosting algorithms confirmed the superior performance of the ANN models, and an explicit neural-network-based correlation was formulated for direct engineering use. Results show that Model A achieved an R² of 0.96 and Model B achieved 0.89, demonstrating that input reduction only slightly reduced predictive accuracy. Both models successfully captured nonlinear relationships, confirming that injection rate, perforation orientation, and perforation number are the most critical factors influencing sand settling. To further test their robustness, cross-validation was carried out using independent experimental data from the literature. Model A achieved an R² of 0.82 and Model B achieved 0.75, showing that both models generalized well to independent datasets, with Model A slightly outperforming Model B. This study provides a novel machine-learning approach for predicting proppant settling in horizontal wellbores under fracturing conditions from experimental data. In contrast to empirical models, the ANN-based predictor accounts for multivariable nonlinear interactions and can be deployed for real-time decision support. The findings contribute to enhanced hydraulic fracturing designs, improved wellbore stability, and reduced operational challenges related to sand production and equipment erosion. Overall, the ANN-based models provide quick and reliable predictions of sand settling, outperforming traditional empirical approaches and offering practical tools for optimizing hydraulic fracturing designs. By accounting for nonlinear interactions and validating independent data, the models demonstrate strong potential for real-time decision support, improved wellbore stability, and reduced operational challenges related to sand production and equipment erosion.

## Introduction

**Fig. 1:**
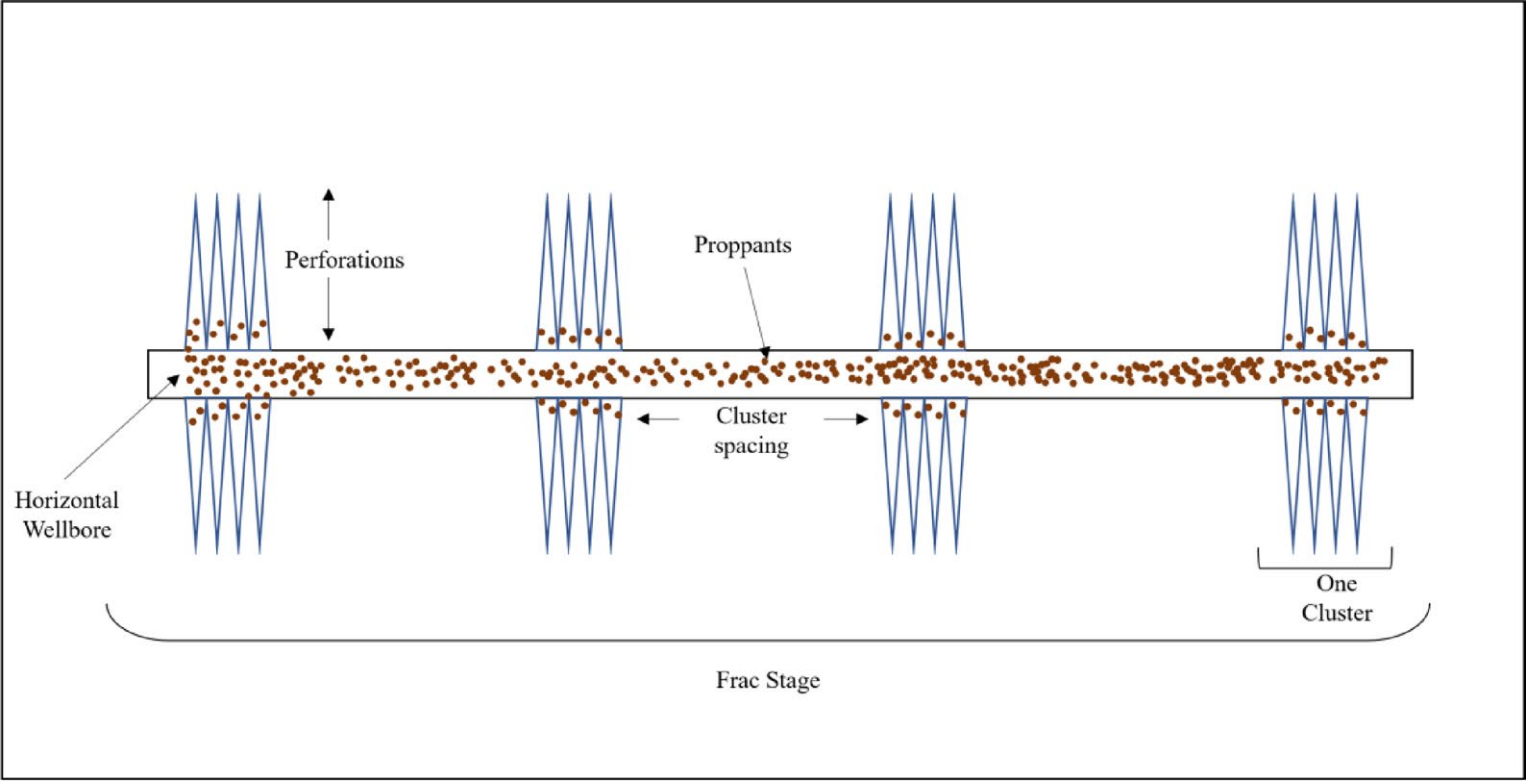
Schematic of a horizontal wellbore with hydraulic fracturing stages. each frac stage contains multiple perforation clusters, with fractures initiated from each cluster.

Achieving uniform proppant placement in multistage horizontal well fracturing is critical for maximizing production in unconventional reservoirs. In plug-and-perf completions, each stage has multiple perforation clusters where slurry exits into fractures, as illustrated in Fig. 1. Ensuring even distribution of proppant across these clusters is challenging but essential, since uneven proppant distribution can significantly reduce fracture conductivity and thus well productivity^1^. Proppant that settles in the horizontal section due to gravity or uneven flow splits can lead to partial cluster plugging and inefficient fracture stimulation. Once pumping stops, any sand accumulated in the wellbore may later be carried to the surface during flowback and production, causing sand production issues. The produced sand can erode surface and downhole equipment, damage valves, and create wellbore blockages, ultimately reducing production rates^2,3^. Field reports and experimental studies indicate that settled proppant in horizontal wellbores is often the initial source of produced sand, which provokes equipment wear and requires costly remediation^3,4^. Therefore, accurately predicting sand settling and proppant distribution in horizontal wells is of great operational importance for designing effective fracture treatments and mitigating post-frac production problems.

Proppant transport and settling in a horizontal wellbore depend on many factors, including wellbore geometry, perforation design, fluid properties, and particle characteristics^1,5,6^. For instance, the internal diameter of the casing or test section, the number and orientation of perforation holes per cluster, the slurry injection rate, proppant size and density, and the viscosity of the fracturing fluid all influence how proppant is distributed among clusters and how much falls out of suspension in the horizontal wellbore^7,8^. High-density or large-diameter proppant tends to settle faster under gravity, especially in low-viscosity fluids like slickwater, whereas higher injection velocities and fluid viscosities can help keep particles suspended. The particle settling velocity in laminar flow can be estimated by Stokes’ law, illustrated in Eq. 1:

1 $$\:{v}_{s}=\frac{\left({\rho\:}_{p}-{\rho\:}_{f}\right)g\hspace{0.17em}{d}^{2}}{18{\mu\:}_{f}}$$

Where $\:{v}_{s}$ is the terminal settling velocity of a spherical particle, $\:{\rho\:}_{p}$ and $\:{\rho\:}_{f}$ are the proppant and fluid densities, $\:d$ is particle diameter, and $\:{\mu\:}_{f}$ is fluid viscosity. This relation indicates that smaller proppants and more viscous fluids result in lower settling velocities, whereas larger particles or less viscous fluids (e.g., water, brine, or nitrogen foam) promote faster settling. In the horizontal well context, reduced gravitational force components and prolonged residence time in the lateral section mean that proppant can accumulate at the bottom of the pipe if flow energy is insufficient to carry it out. Consequently, injection rate (which controls turbulent kinetic energy in the flow) is a dominant factor – higher rates provide greater drag force on particles and more evenly distribute proppant, while low rates can lead to significant gravitational segregation of sand^9^. Field and lab studies have observed that at low viscosity and low flow rate conditions, proppant concentrates toward the toe clusters or settles in the wellbore instead of distributing uniformly^5,6^. These physical insights underscore the need for predictive models that can capture the interplay of variables affecting proppant settling and cluster distribution in horizontal wells.

Many experimental and numerical studies have been conducted to examine proppant transport between perforation clusters and to find methods for improving distribution. Empirical approaches have produced useful correlations, but often under specific conditions. For example, Crespo et al.^10^ performed large-scale inside-casing experiments and highlighted the impact of limited-entry perforations on proppant distribution in multistage horizontal wells. A subsequent study by Zhang and Dunn-Norman^11^ simulations and computational fluid dynamics (CFD) models, respectively, to evaluate proppant distribution across clusters under various phasing and perforation geometries. Their results showed that sand settling and distribution in clusters are influenced by perforation count and phasing and can be optimized via limited-entry designs. Other researchers have explored proppant settling and transport: Gadde et al.^9^ modeled proppant settling behavior in water-fracs, demonstrating the rapid particle settling in low-viscosity fluids; Sahai et al.^12^ investigated proppant transport in complex fracture networks. Notably, Ngameni et al.^13^ developed a laboratory horizontal wellbore apparatus using fresh water to observe proppant transport among clusters, finding that gravity segregation could cause a biased distribution favoring the first clusters and leaving the toe clusters. These empirical and mechanistic studies provided valuable insights and led to the development of semi-analytical correlations for proppant placement. Alotaibi and Miskimins^14^, for instance, derived a scalable correlation for slickwater proppant transport in fractures, and Ahmad and Miskimins^6^ presented new experimental correlations to predict proppant distribution between perforation clusters using low-viscosity fluids in a horizontal wellbore. The latter correlation by Ahmad and Miskimins^6^ used dimensional analysis and laboratory data to relate proppant distribution to key treatment parameters, demonstrating around 85–90% prediction accuracy for cluster-wise proppant split. More recently, Alajmei^1,15^ combined extensive laboratory data (using fresh water with 100 mesh and 40/70 mesh sands) to develop an improved dimensionless correlation for proppant distribution in a horizontal wellbore stage. Using the Buckingham π-theorem, the study incorporated variables such as injection rate, proppant concentration, pipe diameter, fluid viscosity, proppant density, number of perforations, and perforation orientation into a single predictive formula. The resulting correlation could forecast the percentage of proppant allocated to each cluster with a multiple correlation coefficient of about 0.90, offering practical engineering tools. However, as noted by Gupta et al. and Ibrahim et al.^16,17^ such empirical models may not fully capture nonlinear couplings between parameters and can lack generalizability beyond experimental design limits.

In parallel with empirical and physics-based efforts, machine learning (ML) and artificial intelligence techniques have emerged as powerful tools for predicting complex outcomes in oil and gas operations. Unlike traditional models that require explicit physical equations, ML algorithms can learn patterns directly from data, making them well-suited for systems with multiple coupled variables and nonlinear effects^18–20^. In particular, artificial neural networks (ANNs), which are computational models inspired by the human brain, have been widely applied for pattern recognition and regression tasks in petroleum engineering^21–23^. ANNs consist of layers of interconnected artificial neurons that apply weighted sums and nonlinear activation functions to approximate arbitrary functions. Once trained on historical data, an ANN can infer the output for new inputs, effectively capturing relationships that might be intractable to derive analytically^23^. ANNs have already been used for forecasting production, classifying lithology from logs, and estimating reservoir properties^24–26^. In hydraulic fracturing, ANN-based models have predicted flowback control^27^, proppant settling velocity^23^, and essential features affecting transport dynamics^5^.

Despite this progress, few studies have employed ANNs specifically to predict proppant settling within horizontal wellbores using lab data. The present study aims to fill this gap by leveraging a comprehensive experimental dataset and machine learning to forecast sand settling percentages in a horizontal wellbore under various fracturing conditions. The approach is distinguished from past work in that it does not assume a predefined correlation form; instead, the ANN learns directly from the data relationships among nine input parameters. This allows the model to capture subtle nonlinear effects (such as the combined influence of perforation angle and flow rate) that empirical equations might miss.

This study introduces a novel ANN-based predictive framework for sand settling in horizontal wells, with the goals of improving proppant placement design and mitigating sand production. The framework considers nine input parameters, including proppant diameter, injection rate, injected proppant concentration, proppant concentrations at three perforation clusters, valve concentration, perforation orientation, and the number of perforations, with the sand settling percentage serving as the prediction target. Compared to available empirical and semi-analytical models, the proposed ANN approach offers three main advantages: it captures nonlinear interactions among multiple parameters without requiring predefined correlations, it generalizes well when applied to independent datasets as confirmed through cross-validation, and it is trained on experimental laboratory measurements rather than outputs from CFD simulations, ensuring closer alignment with real physical behavior. The following sections present a detailed description of the experimental setup and data collection, the methodology for data analysis and ANN model development, the results of model performance evaluation (including additional error metrics and distributions), and discussions on the model’s insights.

## Methodology and experimental setup

This study employed a laboratory-scale experimental apparatus, originally developed by Alajmei^1^ to generate the dataset used for training and validating the ANN models. The setup was designed to replicate a horizontal wellbore environment under controlled conditions, enabling systematic investigation of how perforation geometry, injection rate, and proppant properties influence transport and settling behavior. By providing controlled and repeatable measurements of proppant distribution across perforation clusters and within the wellbore, the experimental program ensured that the ANN models were trained on data that reflect realistic physical mechanisms rather than purely simulated outputs.

### Experimental setup and data collection

**Fig. 2:**
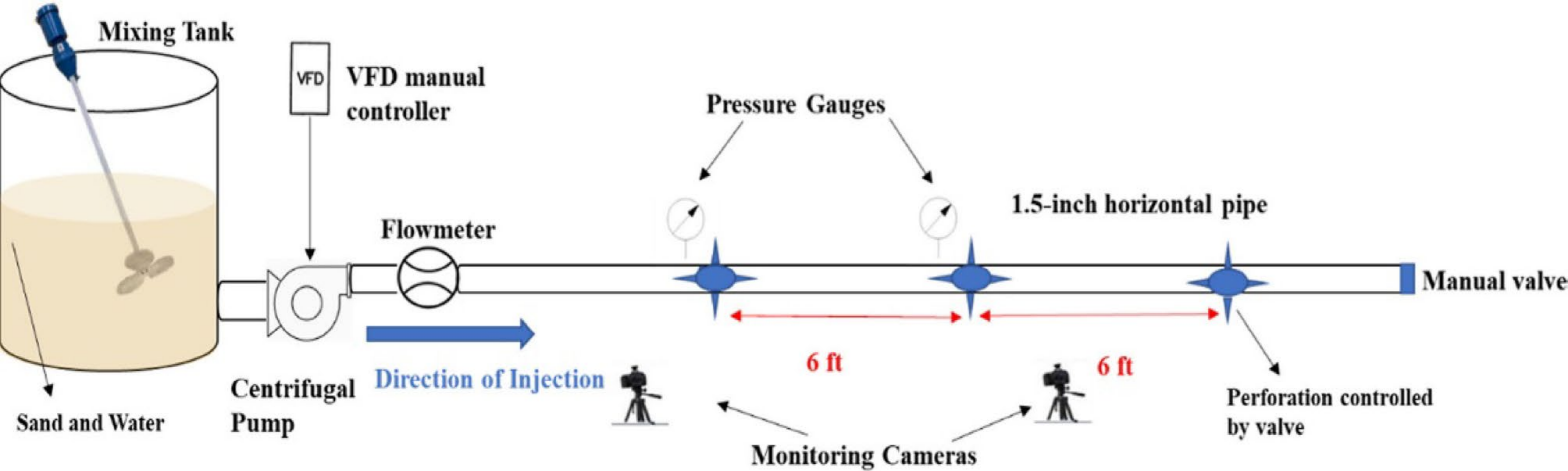
Schematic of the horizontal wellbore experimental setup with three perforation clusters along a 30 ft transparent pipe^1^.

The laboratory experiments were designed to simulate a horizontal wellbore section with multiple perforation clusters and to observe proppant transport and settling under controlled conditions. Figure 2 provides a schematic of the experimental apparatus adapted from Alajmei^1^. The main component is a horizontal transparent pipe, 30 ft in length with an inner diameter of 1.5 in, representing a horizontal wellbore. Along this pipe, three perforation clusters are installed, spaced roughly 7 ft apart to mimic the distribution of perforations in a typical plug-and-perf stage. Each cluster spans a 1 ft section of the pipe and contains a configurable number of perforation holes. The perforations are 0.25 in in diameter, and different cluster designs were tested, ranging from 1 to 4 shots per foot (1–4 SPF) and with various phasing orientations (from 0° up to 180° between perforation holes around the pipe circumference). These variations in perforation density and phasing were introduced to evaluate how cluster geometry affects proppant distribution and settling. A small adjustable valve was installed at the downstream end of the horizontal pipe (toe end) to control the outlet boundary condition. In most tests, the valve was partially opened to allow a limited flow out of the end, simulating the presence of a near-plug or toe valve that permits some fluid and proppant to carry on to the end of the wellbore. In other words, proppant could either exit through one of the perforation clusters or travel to the end valve, similar to field conditions where some slurry may reach the stage tail.

For each experimental run, a slurry of water and proppant was pumped through the horizontal pipe at a controlled injection rate. Fresh water was used as the base fluid (viscosity 1 cP, density 1.0 g/cc) to represent a slickwater fracturing treatment, which is a worst-case scenario for proppant settling due to minimal viscosity support^1^. Two proppant sizes were tested: 100 mesh brown sand and 40/70 mesh white sand (both with specific gravity 2.65). The 100 mesh sand (approximately 150 μm mean diameter) and the 40/70 mesh sand (300–420 μm range) allowed examination of particle size effects on transport; the finer sand was expected to stay suspended more easily than the coarser sand. Proppant was introduced at various concentrations by weight (or in oilfield units, in pounds of proppant per gallon of fluid, ppg). In these experiments, the injected proppant concentration ranged roughly from 0.1 to 4.2 ppg (which corresponds to about 12 to 500 kg/m³). The slurry injection rates were varied between approximately 18 and 76 gallons per minute (gpm) across different tests. This range covers low to relatively high flow conditions for a laboratory pipe of this diameter, ensuring both settling-prone and nearly fully suspended flow regimes are represented. Other fluid and proppant properties, such as proppant density (2.65 g/cc for quartz sand) and fluid density (1.0 g/cc for water), remained constant throughout the tests.

During each run, proppant output from each cluster and from the pipe end was measured to determine how the injected sand distributed itself. The apparatus was equipped with collection containers at each perforation cluster exit and at the end valve. As the slurry was pumped, proppant-laden fluid either jetted out through the perforation holes into the cluster outlets or continued to the pipe end. After a fixed pumping duration (ensuring a known volume of slurry was injected), the pumping was stopped. The fluid and sand captured from each cluster’s outlet and from the end valve were then weighed and analyzed. The proppant concentration at each outlet was calculated (in ppg or equivalent units) by measuring the mass of dry sand collected and the volume of fluid produced at that outlet. Summing the sand from all outlets and comparing with the total sand injected allowed calculation of any sand that might have settled and remained in the pipe. In practice, the settled sand (retained inside the horizontal pipe) was determined by subtracting the recovered proppant mass (all clusters + end) from the injected proppant mass. This ratio was expressed as the sand settling percentage, which is the target output variable for the predictive model. For example, if 10 lb of proppant were injected and 8 lb were recovered from the outlets, the remaining 2 lb in the pipe corresponds to a 20% sand settling percentage for that experiment. Each test thus yielded one data record consisting of the input parameters (injection rate, proppant size, proppant concentration, perforation count, and phasing, etc.) and the measured outcome (sand settling %). In 100 experimental runs were conducted to build a dataset spanning a wide range of conditions. The data collection process ensured consistent measurement procedures for all runs, and the apparatus was cleaned between experiments to remove any residual sand.

The compiled experimental dataset provided a comprehensive basis for analyzing how different factors affect proppant settling in a horizontal wellbore. Key controllable variables (injection flow rate, proppant properties, and perforation configuration) were systematically varied, while environmental conditions (fluid type, pipe size) were kept consistent. This approach enables a focused study on the relative influence of each factor. Notably, the combination of low-viscosity fluid and horizontal geometry in these tests creates a rigorous scenario for proppant transport, where gravitational settling is prominent. The resulting data captured instances of both uniform proppant distribution (e.g., at high flow rates and limited-entry perforations) and severe uneven distribution with significant settling (e.g., at lower flow rates or with many open perforations). This rich dataset is suitable for developing and training an ANN model, as it contains both the extremes and intermediate behaviors of the system. In the next section, we describe how the data were analyzed and prepared, and how the ANN models were developed to predict the sand settling percentage based on the input parameters.

In this study, nine input parameters were considered in developing the ANN models: proppant diameter, actual injection rate, injected proppant concentration, proppant concentrations at Cluster 1 (heel), Cluster 2 (middle), Cluster 3 (toe), and at the outlet valve, perforation orientation angle, and the number of perforations per cluster. The output parameter was the sand settling percentage, defined as the fraction of injected proppant that remained deposited within the horizontal wellbore. The proppant diameter (d, in) denotes the average grain size of the proppant, which directly influences settling velocity as larger particles settle more rapidly under gravity. The actual injection rate (Q, gpm) represents the pumping rate of the fracturing fluid and is a dominant factor controlling suspension and transport; higher flow rates reduce sand accumulation by enhancing drag forces. The injected proppant concentration (Cp, ppg) defines the amount of proppant per unit fluid volume, thereby altering slurry density and viscosity and influencing particle transport behavior.

Cluster-specific measurements were also included to capture proppant distribution along the wellbore. The concentration at Cluster 1 (Cc1, ppg) near the heel reflects the initial exit pattern, while the concentrations at Cluster 2 (Cc2, ppg) and Cluster 3 (Cc3, ppg) represent mid- and toe-side distribution, respectively, serving as indicators of transport efficiency and uneven settling. The concentration at the outlet valve (Cv, ppg) quantifies the fraction of sand bypassing perforations and exiting at the wellbore end, providing additional insight into transport losses.

Geometrical parameters were also considered, including the perforation orientation angle (θ, °), which describes the angular positioning of perforations around the pipe circumference and affects gravitational segregation and flow pathways, and the number of perforations per cluster (N, count), which defines the available flow area and influences slurry distribution and proppant placement. The output variable of interest was the sand settling percentage (Sp, %), defined as the fraction of injected proppant mass that remains deposited within the horizontal wellbore. This parameter served as the primary prediction target for the ANN models, providing a quantitative measure of sand management efficiency under varying injection and perforation conditions.

### Data analysis and ANN model development

In this study, an Artificial Neural Network is used to predict the sand settling in a horizontal borehole after hydraulic fracturing of a horizontal well. The method involves combining and refining two distinct ANN models, based on comprehensive data analysis and a tailored algorithmic strategy.

### Data analysis and preprocessing

The first step of the methodology was to perform a statistical analysis and preprocessing of the experimental data to ensure quality inputs for the ANN. All collected data records were compiled, and obvious outliers or erroneous entries were removed. The remaining dataset was then examined to understand the range and distribution of each variable.

To evaluate the influence of each input variable on the output, the Pearson correlation coefficient between each input and the sand settling percentage was calculated. This correlation analysis helps quantify the strength and direction (positive or negative) of the linear relationship between individual inputs and the target.

The correlation results were used to inform a reduction in input dimensionality. We decided to develop two ANN models: one using all 9 input parameters, and another using a subset of 5 inputs that were deemed most relevant. The criteria for selecting the reduced inputs included: (1) high absolute correlation with the output, (2) known physical significance on sand settling, and (3) the practicality of measuring that parameter in real operations. Based on these criteria, the chosen five inputs for the reduced model were: actual injection rate, proppant concentration at cluster 2, proppant concentration at cluster 3, number of perforations (N), and perforation angle. These variables had relatively higher correlation coefficients and are among the primary factors influencing settling (injection rate being dominant, and the cluster outflow measurements serving as proxies for upstream cluster effects). In excluding the other four inputs (proppant diameter, injected concentration, cluster 1 concentration, and valve concentration), we acknowledge that some information is lost; however, those excluded variables had lower direct correlations and in some cases are inherently linked with the included ones (for example, cluster 1 concentration often varies inversely with cluster 2 and 3 concentrations due to mass balance of sand among clusters). By reducing inputs, we aim to create a simpler model that generalizes better and is easier to deploy (since not all variables may be readily available or needed for prediction). Before training the ANN, the dataset was randomly split into a training set (approximately 70% of the data points) and a testing set (the remaining 30%). The split was done using a randomization function (in MATLAB) to ensure no ordering bias; this means the data points used for testing are essentially a random subset that the model has not seen during training. The use of a separate test set allows for an unbiased evaluation of the model’s predictive performance on new data.

### ANN model development

With the data prepared, we constructed an artificial neural network to map the input parameters to the sand settling percentage. The chosen model type was a feed-forward multi-layer perceptron (MLP), which is a standard ANN architecture for regression problems. In an MLP, neurons are arranged in layers: an input layer (taking our parameters), one or more hidden layers that learn intermediate representations, and an output layer that produces the prediction (sand settling %). Each neuron performs a weighted sum of its inputs and passes the result through a nonlinear activation function. Mathematically, for a simple network with one hidden layer, the model can be expressed as in Eq. 2:

2 $$\:y=f\left(\sum\:_{j=1}^{m}{w}_{j}^{\left(2\right)}\hspace{0.17em}g\left(\sum\:_{i=1}^{n}{w}_{ji}^{\left(1\right)}{x}_{i}+{b}_{j}^{\left(1\right)}\right)+{b}^{\left(2\right)}\right)$$

Where $\:{x}_{i}$ (for i = 1…n) are the input features, $\:{w}_{ji}^{\left(1\right)}$ are weights connecting input i to hidden neuron j, $\:\left(f\right)$ is the hidden layer activation function, $\:{w}_{j}^{\left(2\right)}$are weights from hidden neuron j to the output, and $\:\left(\text{g}\right)$ is the output activation (in our case linear function, since this is regression). The terms $\:{b}_{j}^{\left(1\right)}$ and $\:{b}^{\left(2\right)}$ are bias terms for the hidden and output layer, respectively. It is important to note that the superscripts in Eq. (2) do not represent powers but rather indicate the layer of the network to which the parameters belong. The activation function used in the hidden layer was a sigmoid (logistic) function for Model A, as it provided good performance in initial trials (sigmoid activations are bounded and help the network learn nonlinear relationships).

**Fig. 3:**
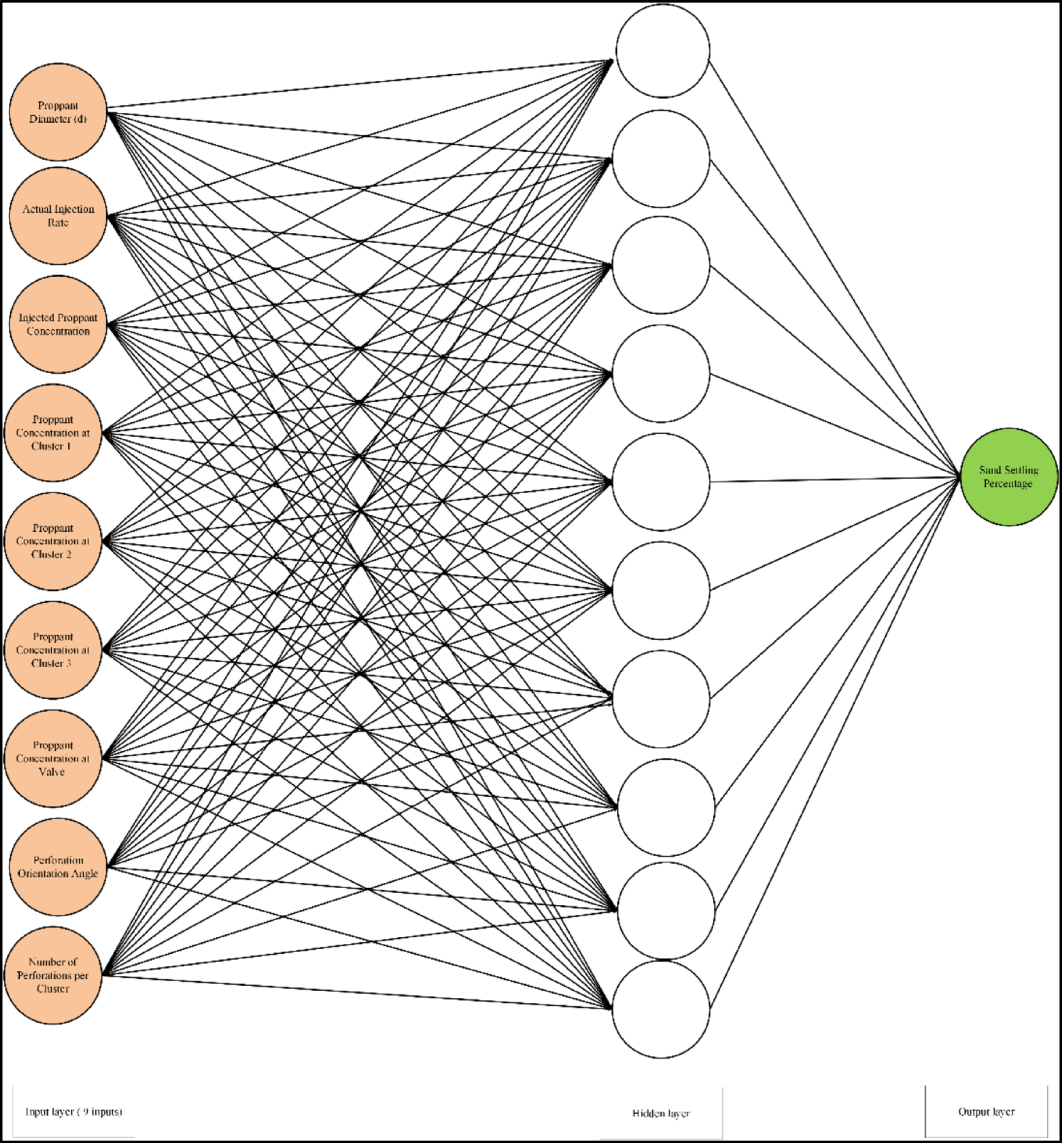
Artificial neural network architecture used for model A (9 inputs, 1 hidden layer with 10 neurons, and a single output).

**Fig. 4:**
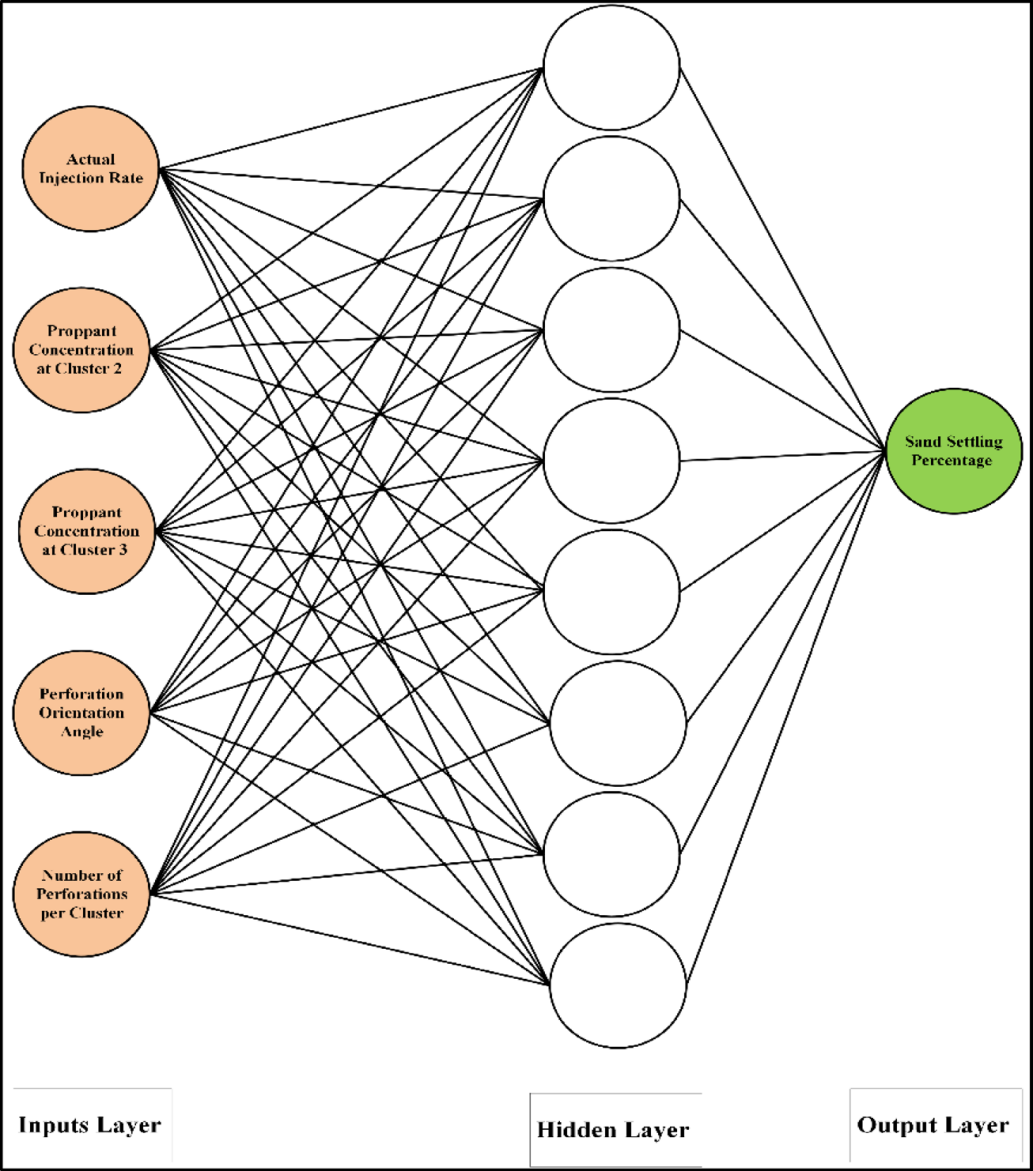
Artificial neural network architecture used for model B (5 inputs, 1 hidden layer with 8 neurons, and a single output).

We experimented with different network topologies to find an optimal design. The number of hidden layers and the number of neurons in each layer were varied, as was the choice of training algorithm (backpropagation variants) and transfer functions. Using a trial-and-error approach guided by performance on a validation subset of the training data, we identified a suitable architecture. The final ANN architecture for both Model A and Model B consisted of a two-layer network (one hidden layer and one output layer). Model A (with 9 inputs) used a hidden layer of 10 neurons as illustrated in Fig. 3, while Model B (with 5 inputs) used a hidden layer of 8 neurons as illustrated in Fig. 4. These sizes were found to minimize the error without overfitting. Both models used a Levenberg–Marquardt backpropagation training algorithm (a fast converging second-order method well-suited for moderate-sized datasets) to optimize the weights. The training objective was to minimize the mean squared error (MSE) between the network’s predicted settling percentage and the actual observed percentage from experiments. The MSE function can be written as in Eq. 3:

3 $$\:MSE=\frac{1}{N}\sum\:_{k=1}^{N}{\left({y}_{k}^{pred}-{y}_{k}^{obs}\right)}^{2}$$

where $\:N$ is the number of training samples, and $\:{y}_{k}^{pred}$ and $\:{y}_{k}^{obs}$ are the predicted and observed settling values for sample $\:k$. The weight vectors $\:\text{w}$ (comprising $\:{w}^{\left(1\right)},{w}^{\left(2\right),}$ and biases) are iteratively updated in the direction of decreasing $\:MSE$. The weight update rule for a simple gradient descent would be $\:{\text{w}}_{new}={\text{w}}_{old}-\eta\:\hspace{0.17em}{\nabla\:}_{\text{w}}E,$ where $\:\eta\:$ is the learning rate. In Levenberg–Marquardt, the update is refined by approximating second-order curvature, which accelerates convergence. Training was carried out until the error plateaued and further iterations yielded negligible improvement. We employed early stopping by monitoring error on a validation subset: training was halted when validation error began to rise (to avoid overfitting).Two ANN models were developed in this study to evaluate the effect of input dimensionality on predictive performance. Model A incorporated all nine input parameters, thereby capturing the full complexity of the dataset. Model B, by contrast, was constructed using only five selected inputs that showed the highest correlation with the output variable, reducing model complexity while retaining the most influential parameters.

Model A was trained with all nine inputs, capturing the full complexity of the data. Model B was trained separately with the five selected inputs. During training, it was observed that Model A, with more inputs and degrees of freedom, achieved a very low training error but was at slight risk of overfitting (the gap between training and validation error had to be monitored). Model B, being simpler, trained faster and tended to generalize slightly better on unseen data, although its minimum training error was higher than Model A’s (since it had less information to work with). In both cases, we adjusted the network hyperparameters (like hidden neurons) to strike a balance between underfitting and overfitting. The final chosen models were those that yielded the lowest testing error while maintaining reasonable complexity.

After training the ANNs, we evaluated their performance using several metrics. The primary metrics reported are the coefficient of determination (R²) and the Average Absolute Difference (AAD), Mean Squared Error (MSE), and Mean Absolute Percentage Error (MAPE). R² is defined as in Eq. 4:

4 $$\:{R}^{2}=1-\frac{\sum\:_{k}{\left({y}_{k}^{pred}-{y}_{k}^{obs}\right)}^{2}}{\sum\:_{k}{\left({y}_{k}^{obs}-{\bar{y}}^{obs}\right)}^{2}}$$

Which represents the proportion of variance in the observed data explained by the model(with $\:\stackrel{-}{{y}^{obs}}$ being the mean observed settling). An $\:{R}^{2}$ of 1 indicates a perfect fit, whereas 0 indicates the model is no better than using the mean. The AAD is defined here as the mean of the absolute differences between predicted and observed values, as shown in Eq. 5:

5 $$\:\text{AAD}=\frac{1}{N}\sum\:_{k=1}^{N}\left|{y}_{k}^{pred}-{y}_{k}^{obs}\right|$$

In essence, AAD is equivalent to the mean absolute error (MAE) in this context, expressed in percentage points of sand settling. While AAD/MAE gives an average magnitude of errors, we also computed the mean squared error (MSE) and the mean absolute percentage error (MAPE) for a more comprehensive evaluation. MAPE (here also referred to as AAP – Average Absolute Percentage error) is calculated with Eq. 6:

6 $$\:\text{MAPE}=\frac{100\text{\%}}{N}\sum\:_{k=1}^{N}\left|\frac{{y}_{k}^{pred}-{y}_{k}^{obs}}{{y}_{k}^{obs}}\right|$$

which expresses the error as a percentage of the actual value (averaged over all samples). MAPE is useful to understand the relative error, especially since sand settling percentages in our data span a wide range (an absolute error of 3% is minor if the true value is 50%, but significant if the true value is 5%). In addition to numeric metrics, we generated error distribution plots to visualize the spread of prediction errors. These plots help identify if the model has any bias (e.g. consistently under- or over-predicting) or if there are particular cases with large errors. We also tracked the training and testing errors separately to ensure that the model’s performance generalizes beyond the training data.

It is worth noting that various other machine learning techniques could potentially be applied to this regression problem. Methods such as Random Forests, Support Vector Machines (SVMs), or gradient boosting algorithms can handle nonlinear relationships as well. As shown in Sect. 3.5, we conducted a Comparative Performance with Tree-Based Models but chose to go with ANN for many reasons. First, an ANN is a universal function approximator and was found to be highly suitable for capturing the continuous, nonlinear mapping in our problem without requiring extensive feature engineering. In preliminary trials, simpler models like decision trees or linear models failed to achieve comparable accuracy, likely due to the complex interactions among inputs. Random Forests, while powerful for many problems, generate an ensemble of decision trees that can be less transparent and can require a large number of trees to approximate smooth relationships in continuous data. They also do not extrapolate beyond the range of training data, whereas an ANN with appropriate training can interpolate the underlying functional behavior more smoothly. SVMs with nonlinear kernels could, in theory, fit the data, but they involve tuning of kernel functions and can be computationally intensive as the dataset grows. Moreover, SVMs yield a model that is not as easily interpretable in terms of variable influence without additional analysis (like support vectors or feature weights in the kernel space). In contrast, the ANN approach allowed us to incorporate domain knowledge (by selecting inputs and designing the architecture) and resulted in a straightforward model deployment – once trained, the ANN calculation is fast and can be implemented in real-time monitoring systems. Finally, the research objective was also to demonstrate the applicability of ANNs specifically, as a novel approach in this domain, building on the prior success of ANNs in similar engineering prediction tasks^21,23^. For these reasons, other ML methods were not pursued in this study, and efforts were concentrated on optimizing the neural network models.

The ANN architecture employed in this study shares structural similarities with standard feedforward neural networks, but the design of our model differs in both foundation and application. Each input parameter was selected not only based on statistical correlation but also for its physical relevance to proppant transport behavior—for example, injection rate influencing inertial drag and perforation angle affecting gravity-driven particle segregation. These inputs are grounded in experimental observations rather than simulations, which enhances the physical interpretability of the model outcomes. In contrast to the recent study by Qu et al.^28^, which trained an ANN using outputs from CFD-DEM simulations, our model is built on data generated from controlled laboratory experiments that closely replicate horizontal wellbore conditions. Furthermore, measured outputs such as proppant concentrations at different clusters were directly incorporated as ANN inputs, embedding observability into the model structure. Although the network mechanics are conventional, the input selection and data origin make this approach distinct and provide a foundation for future integration of physics-informed machine learning frameworks.

## Results and discussions

This section presents the outcomes of the study in three stages. First, the dataset is examined through statistical analysis to understand the variability of the input and output parameters. Next, the predictive performance of the two ANN models is evaluated using error metrics and validation tests. Finally, the results are interpreted in terms of proppant transport mechanisms and compared with existing models.

### Statistical analysis and pearson correlation coefficient

A summary of the key input parameters and the output (sand settling percentage), including their minimum, maximum, and selected statistical values, is shown in Table 1. For instance, the actual injection rate in the experiments ranged from about 17.7 gpm up to 76.3 gpm, while the injected proppant concentration ranged from 0.08 ppg to 4.17 ppg. The sand settling percentage observed varied widely from as low as 0.15% (virtually no sand left in the pipe) up to 68.7% (a majority of sand settled). This broad variability confirms that the dataset covers both near-ideal transport conditions and very poor transport scenarios. All inputs (such as flow rate, concentrations, etc.) were normalized to a common scale for ANN training – specifically, inputs were scaled between 0 and 1 based on their observed min–max range. This normalization prevents variables with larger numeric ranges from unduly dominating the ANN weight updates and generally leads to more stable training.

**Table 1:** Summary of the input parameters and the output (sand settling percentage), including their statistical analysis results.

Parameters	Proppant Diameter (in)	Actual Injection Rate (gpm)	Injected Proppant Concentration (kg/m3)	Cluster 1, (ppg)	Cluster 2, (ppg)	Cluster 3, (ppg)	Valve (ppg)	Number of Perforations *N*	Angle (degree)	SettlingP (%)
**Minimum**	0.00019	17.66	0.08	0	0.01	0.01	0	1	0	0.15
**Maximum**	0.00033	76.30	4.17	3.99	2.42	2.31	3.68	4	180	68.67
**Arithmetic Mean**	0.00025	41.77	0.90	0.79	0.59	0.55	0.46	2.08	75.6	9.29
**Harmonic Mean**	0.00023	35.32	0.456	0	0.13	0.11	0	1.59	0	3.16
**Mode**	0.00019	18.32	0.33	0.02	0.79	0.04	0	1	0	2.08
**Range**	0.00015	58.64	4.09	3.99	2.41	2.30	3.68	3	180	68.52
**Variation**	5.4E-09	279.92	0.54	0.68	0.32	0.26	0.45	1.13	5026.91	116.25
**IQR**	0.00015	24.19	0.92	0.94	0.61	0.63	0.57	2	90	7.43
**Standard Deviation**	7.5E-05	16.73	0.73	0.82	0.57	0.51	0.67	1.06	70.91	10.78
**Skewness**	0.242	0.44	1.70	1.75	1.40	1.27	2.43	0.45	0.29	3.01
**Kurtosis**	1.059	2.09	6.72	6.42	4.48	4.11	9.75	1.89	1.68	14.39
**Coefficient of variation**	29.37	40.05	81.78	104.25	96.18	93.39	145.84	50.99	93.78	116.06

**Fig. 5:**
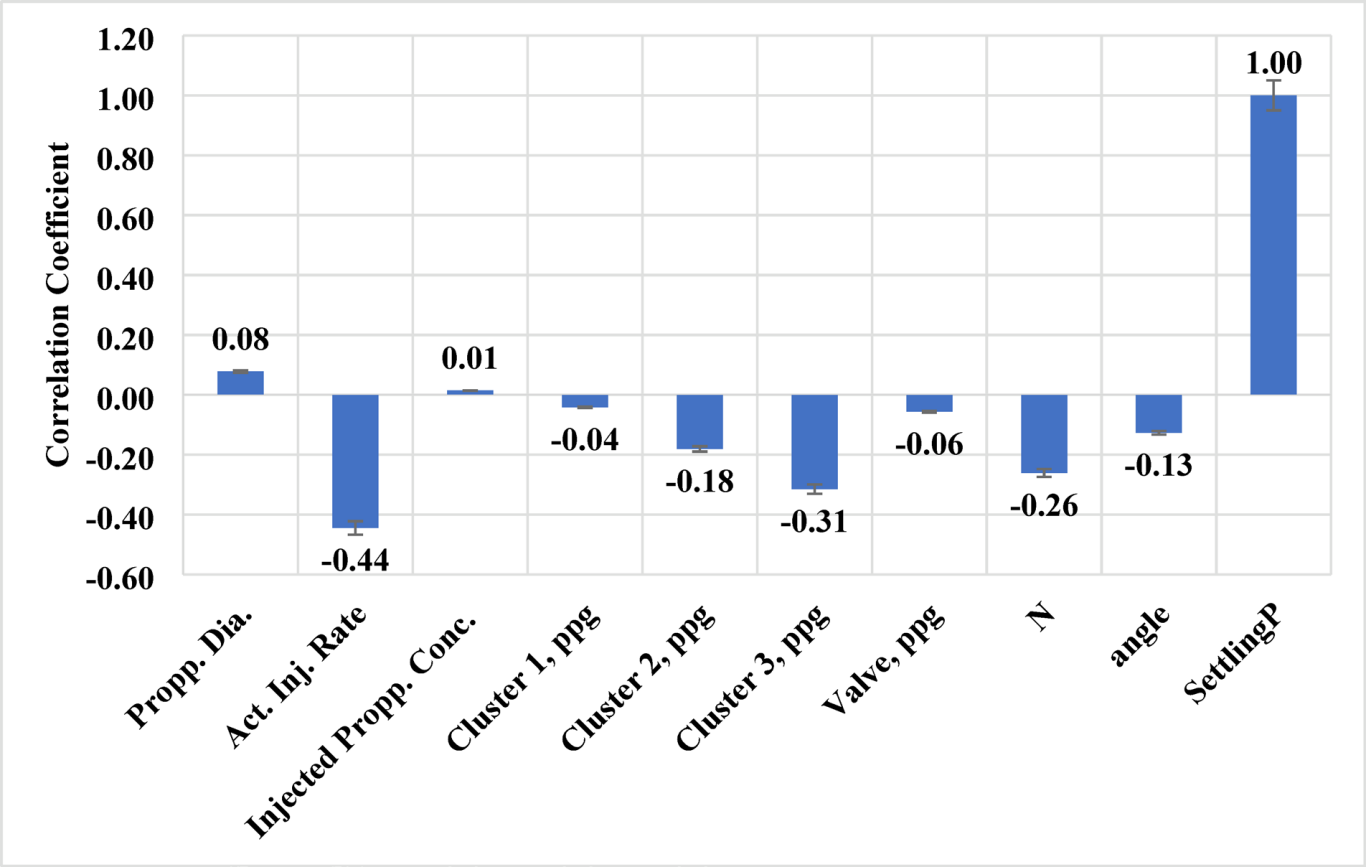
Correlation coefficient results for predicting the sand settlement using different treatment parameters.

The correlation coefficient parameter was calculated to measure the relationship between the input and output variables; the results are illustrated in Fig. 5 (a bar chart of correlation coefficients for all input variables). In our case, the actual injection rate exhibited the highest magnitude correlation with sand settling (with a negative correlation value). This indicates a strong inverse relationship: as the injection rate increases, the sand settling percentage tends to decrease, which is consistent with physical expectations (higher flow rates carry more sand out, so less settles). The number of perforations (N) per cluster also showed a notable negative correlation with sand settling – effectively, using more perforations (larger total flow area) led to more sand exiting into clusters (and less settling in the wellbore) under our test conditions. Similarly, the measured proppant concentrations at the clusters (especially cluster 2 and cluster 3) correlated inversely with settling: higher sand return in those clusters implies less sand remaining to settle. On the other hand, the proppant diameter had a positive correlation with sand settling percentage, meaning larger proppant size promotes more settling (again aligning with expectations, as larger grains are harder to keep suspended). The perforation orientation angle had a weaker positive correlation, suggesting that for our range of phasing (0° to 180°), certain orientations slightly increased settling (likely because certain phasing arrangements favor top or bottom exits, affecting how sand falls out). It should be noted that these correlation coefficients only capture linear pairwise relationships. Some variables may have nonlinear or interactive effects on settling that are not obvious from the linear correlation alone. For example, the effect of injected proppant concentration on settling is complex: very high concentrations might increase settling due to particle-particle interactions, but within our range, the correlation was modest. Therefore, while correlation analysis is useful for initial feature importance screening, the ANN model (which can capture nonlinear interactions) is needed for a more complete understanding.

### ANN model a: full-parameter ANN performance

Sand settling was predicted using artificial neural networks (ANNs) developed with different sets of input parameters. Two models were constructed: Model A, which used all nine available inputs, and Model B, which used a reduced set of five inputs identified through correlation analysis. Both models were optimized by adjusting the number of hidden layers, neurons, training functions, and transfer functions to minimize prediction error.

**Fig. 6:**
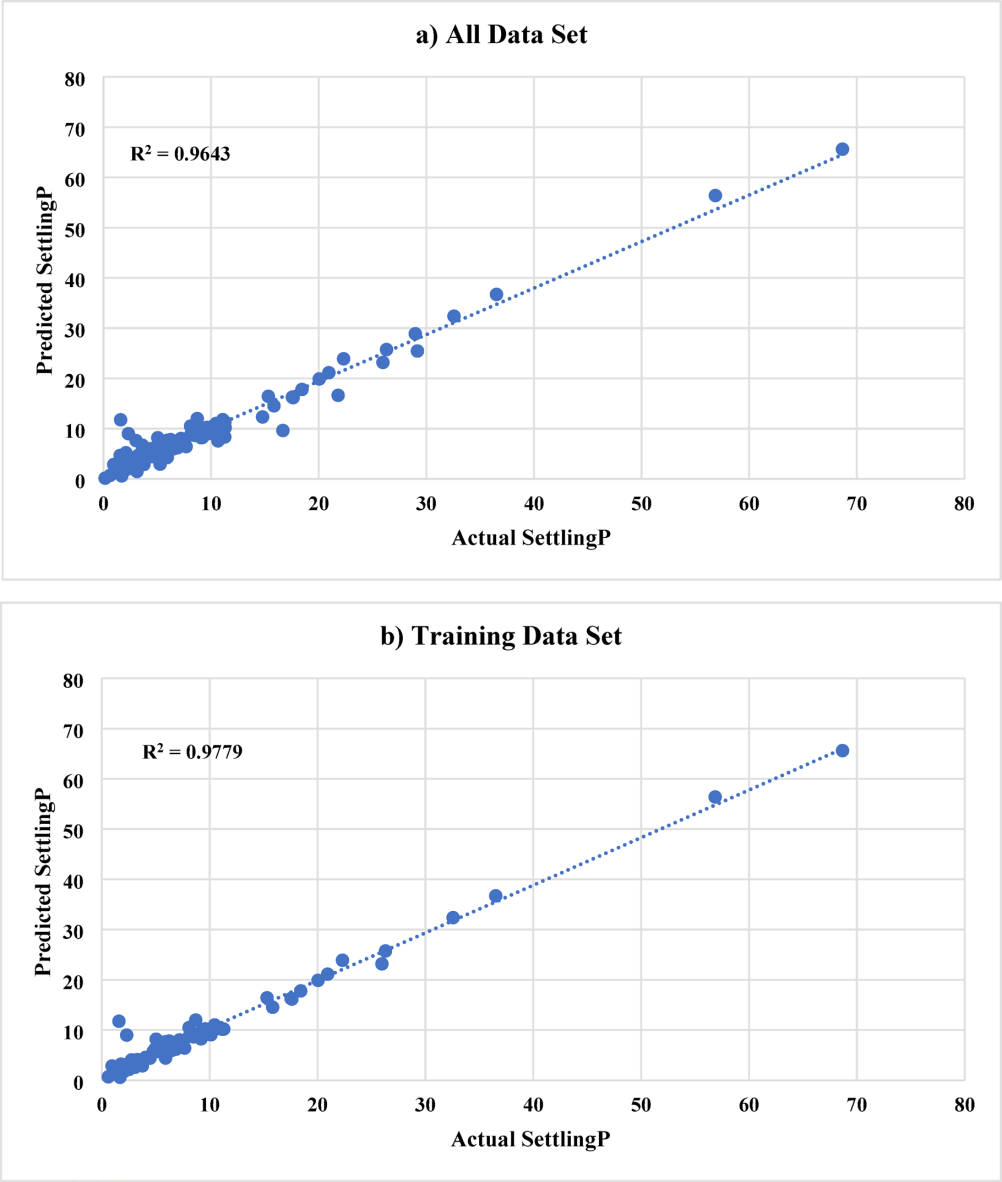

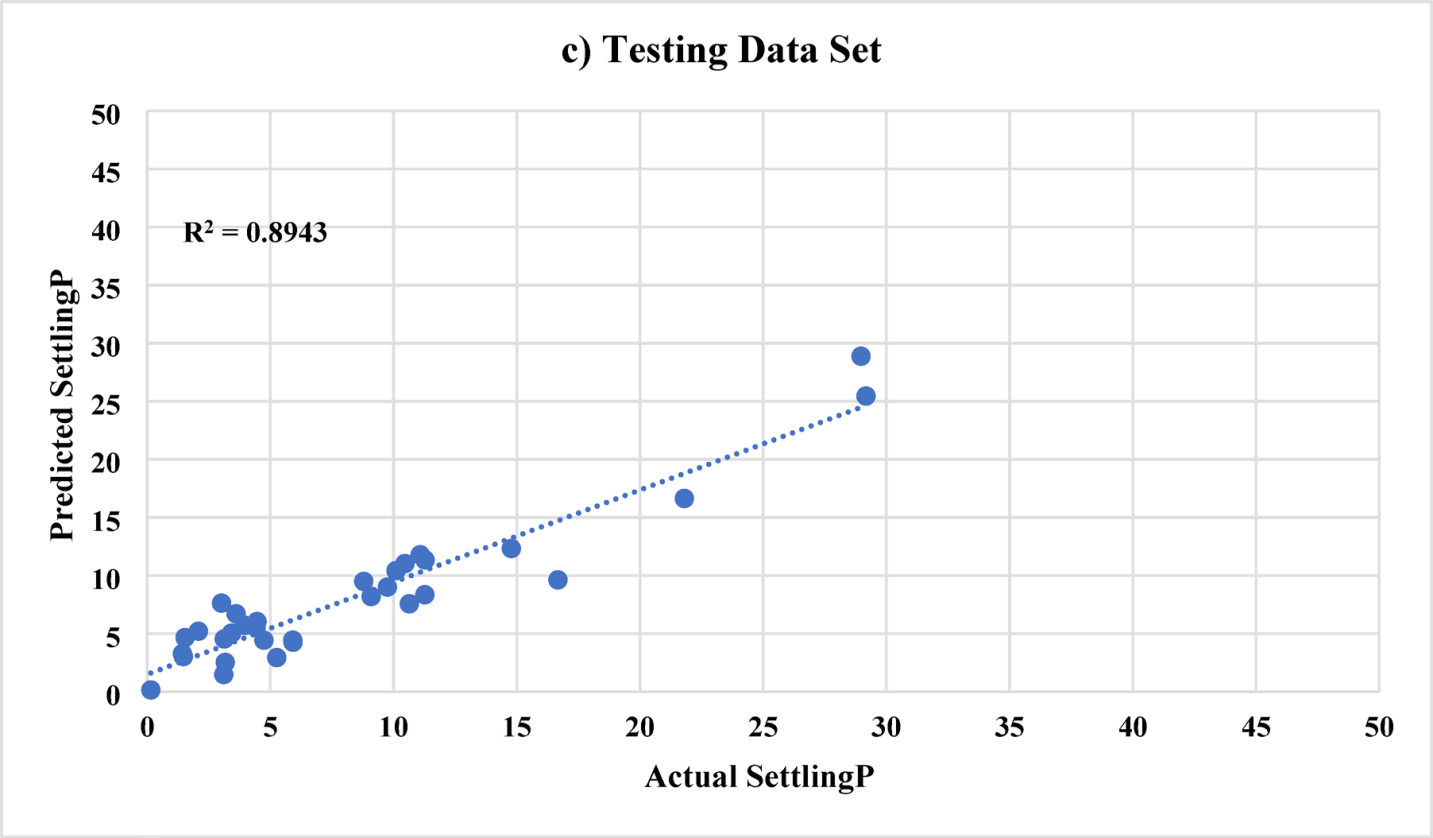
ANN results (Model A) for predicting the sand settlement using **a**) all data set, **b**) training data sets, and **c**) testing data sets, respectively.

In ANN model A, 9 inputs were used to predict the sand settlement. The used inputs are the proppant (sand) diameter, actual injection rate, injected proppant concentration, cluster 1, cluster 2, cluster 3, valve, N, and the angle. Figure 6 shows the ANN results (named as Model A) for predicting the sand settlement using all, training and testing data sets, respectively. The developed model predicts the sand settlement with an R^2^ of 0.96 and an average absolute difference (AAD) of 1.36 using the data set. It should be noted that the whole data was categorized into training data (Around 70%) and testing data (around 30%). The training data set was used mainly to train the ANN model to capture the relationship between the input parameters and the output. During the training phase, the model was able to predict the sand settling percentage with an R^2^ of 0.97 and AAD of 1.10. Then, the model predicts the sand settling for the hidden data set (testing data) with a very acceptable accuracy; R^2^ is 0.89 and AAD is 1.98. It should be highlighted that the training and testing data sets were randomly selected using the Randomization function in MATLAB, which will provide more reliability and reduce the model memorization as well as the data ordering effects.

**Fig. 7:**
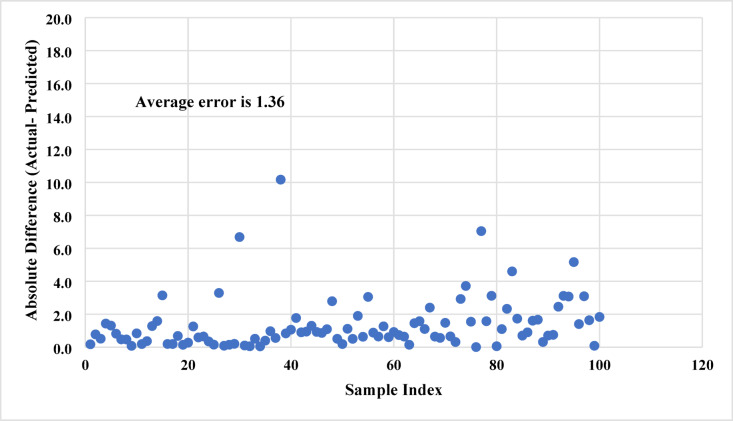
Distribution of absolute prediction errors for model A. more than 90% of the samples show an error below 4%, indicating strong model accuracy and minimal bias across the test set.

Moreover, the error distribution for all considered samples was determined. Figure 7 shows the distribution of the absolute difference for sand settlement prediction using Model A. More than 90% of the studied samples showed an absolute error of less than 4% for sand settlement, ranging from 0.15 to 68.67%. Also, the average absolute difference is 1.36 for all samples, indicating the high reliability of the developed ANN model. However, the main concern for this ANN model (Model A) could be the high number of inputs; therefore, an improved model could be developed to reduce the number of inputs while maintaining a reasonable prediction performance, as will be discussed in the coming sections.

To further evaluate Model A, we also computed additional metrics on the test set: The test MSE was approximately 4.1%, which corresponds to a Root Mean Squared Error (RMSE) of about 2.0% points. This is consistent with the earlier AAD/MAE of 1.98% and indicates a low variance in errors (no very large errors that would inflate MSE significantly). The MAPE on the test set was found to be around 8.5%. This relatively small percentage error indicates that, on average, the model’s prediction deviates from the actual value by less than 10% of that value. For instance, if the true sand settling was 40%, the model might typically predict between 36 and 44%. Taken together, these metrics (high R², low MAE, low MAPE) confirm that Model A provides accurate and consistent predictions across the range of scenarios tested.

One potential drawback of Model A is its complexity: it requires all nine input parameters to be known or measured. While this is not an issue in a controlled experiment, in real field operations, one may not readily have all those inputs (for example, the exact proppant concentration split among clusters might not be measurable in real time without sophisticated downhole sensors). Therefore, a simpler model that still retains good accuracy is desirable. We address this with Model B, discussed next.

### ANN model B: reduced-input ANN performance

**Fig. 8:**
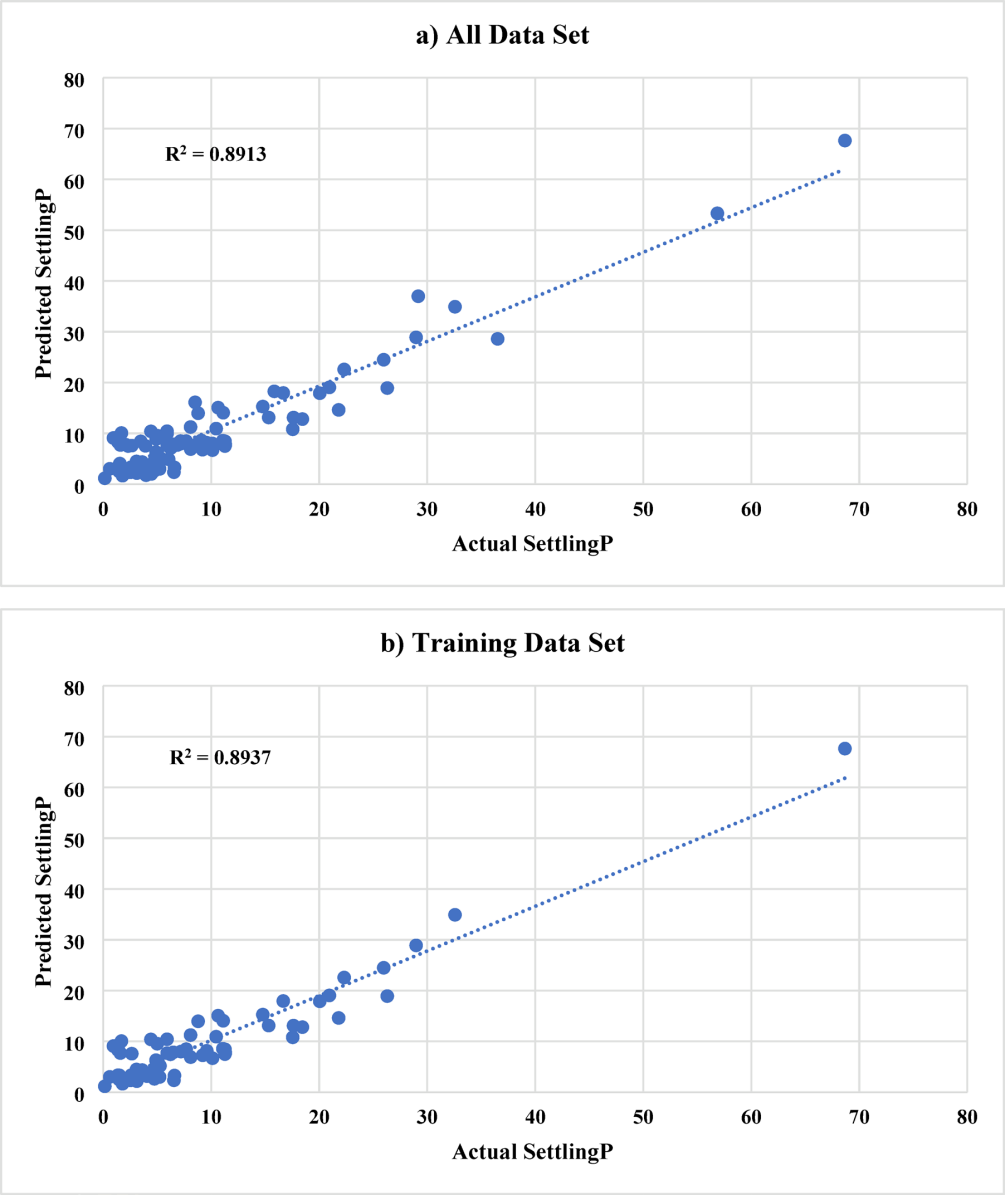

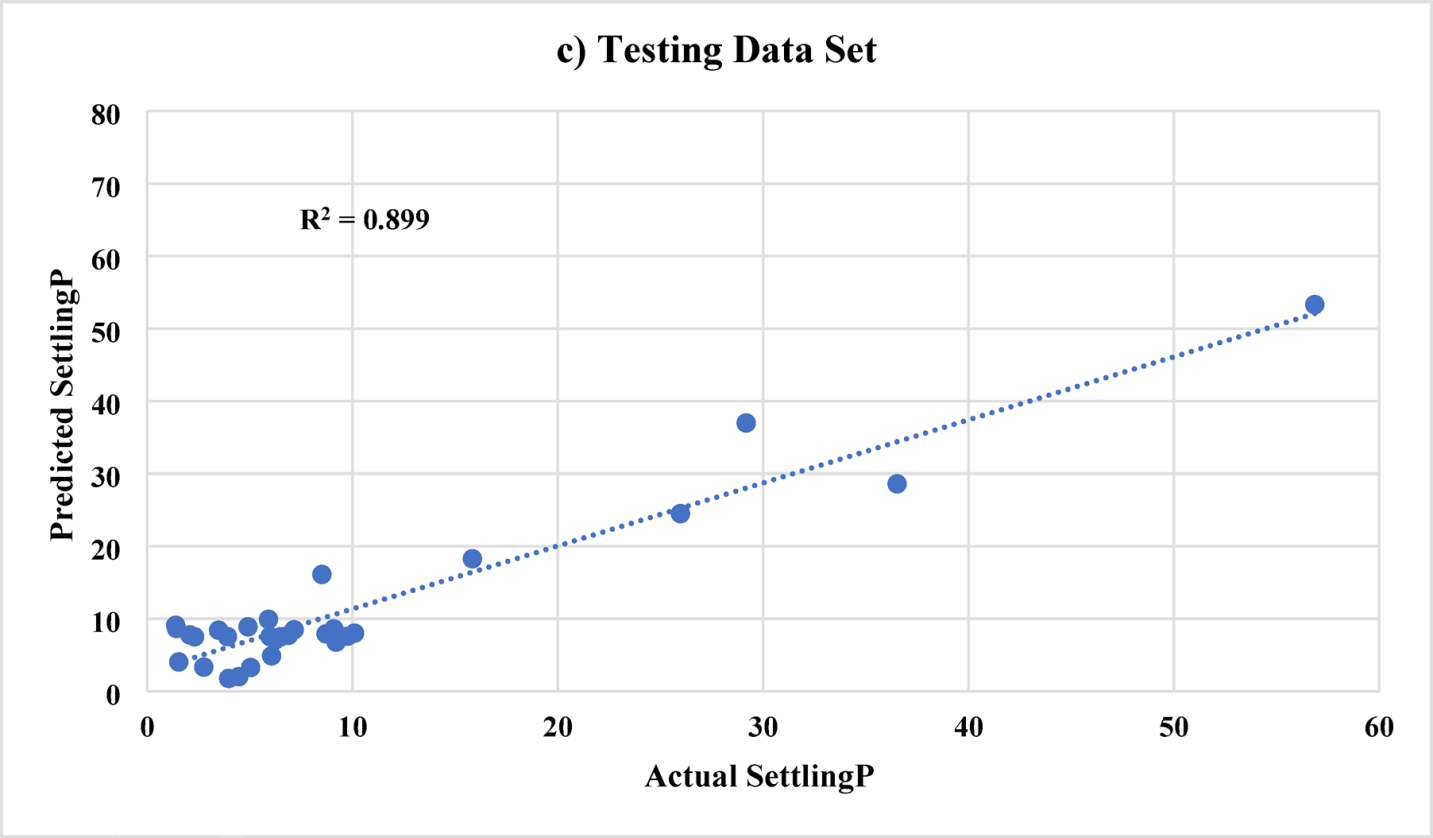
ANN results (Model B) for predicting the sand settlement using **a**) all data sets, **b**) training data sets, and **c**) testing data sets, respectively.

An improved ANN model was developed by reducing the number of inputs from 9 (as in Model A) to 5 (which will be named Model B). The considered inputs are the actual injection rate, cluster 2, cluster 3, N, and the angle. While the proppant (sand) diameter, injected proppant concentration, cluster 1, and valve were excluded this time due to their relatively low correlation coefficient values. It is worth mentioning that 5 inputs were selected for developing Model B; however, the number of inputs could be further reduced by excluding parameters such as cluster 2 and angle due to their small correlation coefficient values. Figure 8 shows the actual sand settlement against the predicted using Model B for all training and testing data sets, respectively. The improved model predicts the sand settling percentage with AAD of 3.02, 2.58, and 2.71 for the testing, training, and all data sets, respectively. The coefficient of determination (R^2^) is more than 0.89 for the three considered data sets. The evaluation indices (AAD and R^2^) indicate that the improved model has very acceptable prediction performance for estimating the sand settlement in the range of 0.15 to 68.7%.

**Fig. 9:**
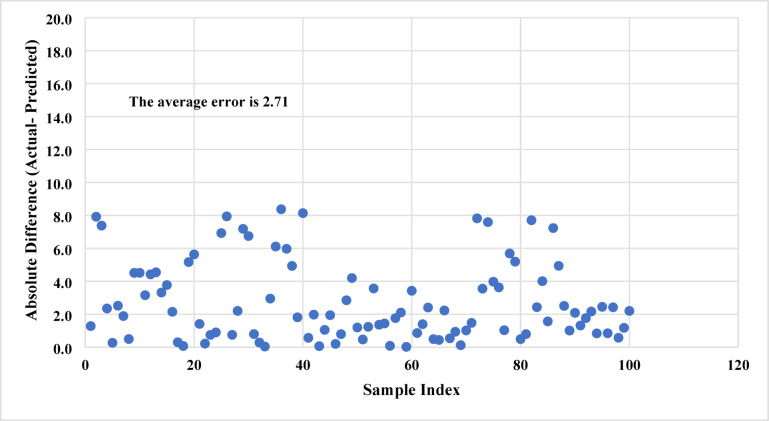
Distribution of absolute prediction errors for model B. Approximately 90% of predictions deviate by less than 6% from experimental values, confirming that the reduced-input model maintains acceptable predictive performance.

In addition, the error distribution for predicting the sand settlement using Model B is shown in Fig. 9**.** Around 90% of the data shows an absolute error of less than 6% while the average absolute difference (AAD) is 2.71, revealing the reasonable prediction performance for the improved ANN model.

For completeness, we also computed Model B’s MSE and MAPE on the test set. The MSE for Model B was approximately 10.4%, corresponding to an RMSE of about 3.22% points. This is higher than Model A’s RMSE (2.0%), as expected. The MAPE for Model B was around 15–16%, indicating that, on average, the prediction error was 15% of the actual value. This is still fairly good, but about twice Model A’s relative error. For example, if the true settling was 40%, Model B might be off by 6% (which is 15% of 40) on average, whereas Model A would be off by 3.4% on average (8.5% of 40). Thus, Model A provides more precise estimates, but Model B’s accuracy might be sufficient for many practical engineering decisions, especially considering it requires fewer inputs.

Table 2 provides a side-by-side comparison of the performance metrics of Model A and Model B on the training, testing, and combined datasets. As discussed, Model A outperforms Model B in terms of absolute error (AAD/MAE) across all datasets. For instance, on the testing set Model A’s AAD was 1.98% vs. Model B’s 3.02%. Model A’s test R² was slightly lower than Model B’s (0.894 vs. 0.899), but this difference is negligible; effectively, both models explain 89–90% of the variability in the test data. The small R² uptick for Model B likely stems from Model A capturing some noise in training that didn’t generalize. In summary, Model A is more accurate but more complex, while Model B is simpler yet still reasonably accurate. Depending on usage, one might choose Model B for its simplicity (if real-time data on all 9 inputs is not available) or Model A for maximum accuracy (if all inputs can be obtained and a precise answer is needed). It is encouraging that Model B retained such high performance with nearly half the inputs removed; this implies that there was some redundancy or lesser importance in the excluded features, validating our feature selection approach via correlation analysis.

**Table 2:** Summary of the ANN model prediction performance for model A and model B.

Data Set	Metric	Model A	Model B
**All**	AAD (%)	1.36	2.71
	R²	0.964	0.892
	MSE	3.52	8.16
	MAPE (%)	5.21	12.64
**Training**	AAD (%)	1.10	2.58
	R²	0.978	0.894
	MSE	2.81	7.23
	MAPE (%)	4.62	11.82
**Testing**	AAD (%)	1.98	3.02
	R²	0.894	0.899
	MSE (%)²	4.10	10.40
	MAPE (%)	8.50	15.80

In both models, most prediction errors are small and symmetrically distributed around zero, indicating the absence of systematic bias. The error-distribution plots (Fig. 7 and **Fig. 9**) show that over 90% of the samples exhibit absolute errors below 4% for Model A and below 6% for Model B. Only a few outlier cases exceeded 10% error, typically corresponding to experimental runs with extreme combinations of very low injection rates and high proppant loadings, where flow behavior departs from the bulk of the dataset.

These outliers have limited influence on the overall model statistics (Table 2) but are important to recognize for operational planning. In hydraulic-fracturing design, an error of ± 5% in predicted sand-settling percentage translates to a small change in the estimated sand hold-up or cluster-efficiency ratio, well within the uncertainty margin of typical field measurements. However, in scenarios approaching flow-back limits or high-deposition risk, even moderate under-predictions could indicate a need for higher injection rates, lower proppant concentrations, or design modifications such as reduced cluster spacing.

Overall, the narrow error distribution and the physically interpretable location of the few high-error points confirm that the ANN models are reliable for routine design optimization. The outliers highlight conditions where the system transitions toward dense-flow or gravity-dominated regimes, providing guidance on the limits of model applicability.

### Sensitivity analysis

Sensitivity analysis was conducted for the input parameters of Model A and Model B.

#### Model A

The impact of the input parameters on the ANN prediction performance was investigated.

**Fig. 10:**
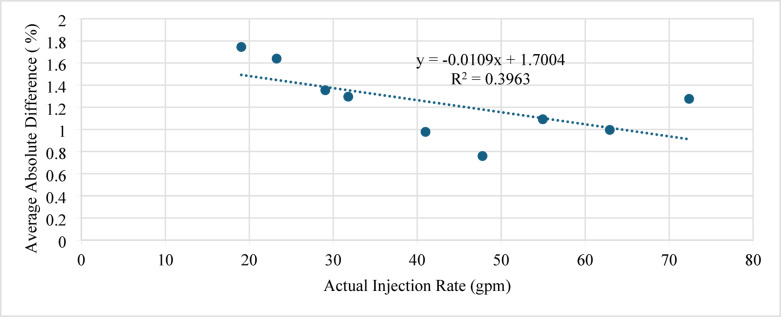
Average absolute difference (AAD) between actual and predicted sand-settling percentage against injection rate.

Figure 10 shows the average absolute difference (AAD) between the actual and predicted sand-settling percentages plotted against the injection rate.

Generally, higher injection rates lead to lower settling and hence smaller prediction errors. In this work, increasing the injection rate from 20 to 70 gpm reduced the AAD from 2.9 to 1.1%, indicating that the ANN model provides more accurate predictions at higher flow rates. However, the percentage error in all cases remained within ± 5%, which is considered acceptable for engineering applications.

**Fig. 11:**
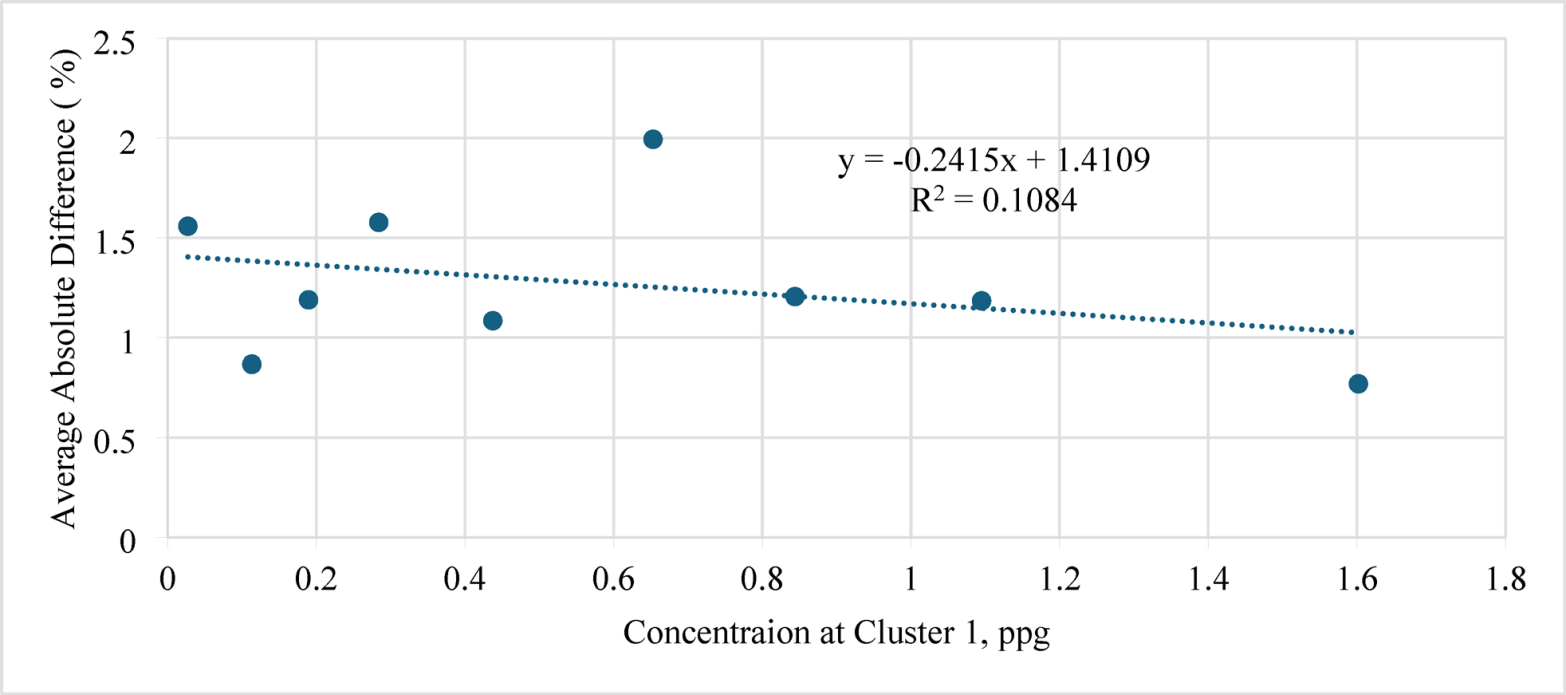
Average absolute difference (AAD) versus concentration at Cluster 1 (ppg).

Moreover, Fig. 11 shows the average absolute difference versus concentration at Cluster 1. Similarly, the AAD decreased slightly from 2.4 to 1.2% with increasing Cluster 1 (ppg), implying that the model better represents the experimental trend when the near-heel cluster receives more proppant.

**Fig. 12:**
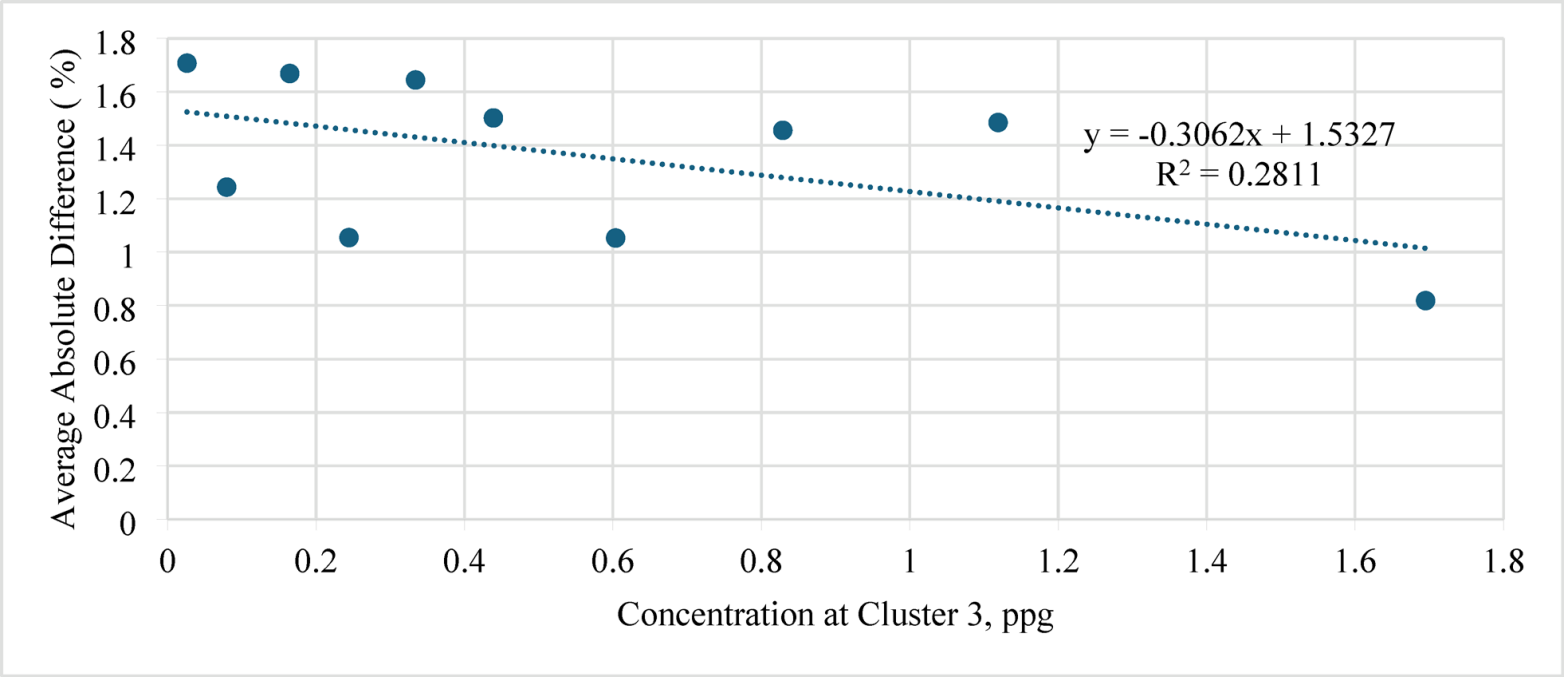
Average absolute difference (AAD) versus concentration at Cluster 3 (ppg).

Figure 12 illustrates the relationship between the average absolute difference and concentration at Cluster 3. As the Cluster 3 (toe-side) concentration increased from 0.3 to 1.5 ppg, the AAD decreased from 2.7 to 1.0%. This indicates that the ANN model captures the improvement in proppant transport toward the toe cluster, which reduces settling and enhances prediction accuracy.

**Fig. 13:**
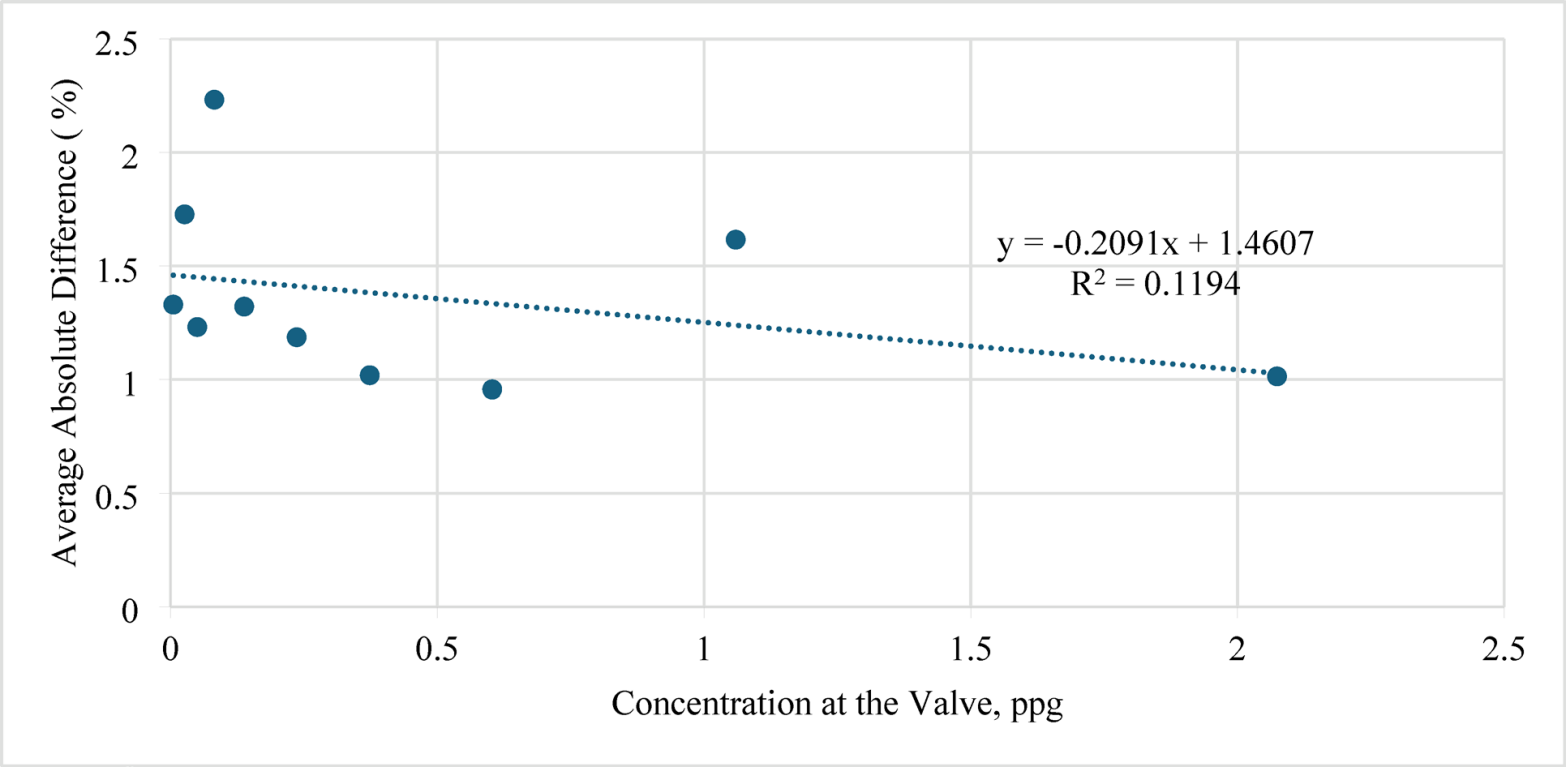
Average absolute difference (AAD) versus concentration at the valve (ppg).

In addition, the profile of the average absolute difference against concentration at the valve is shown in Fig. 13. A gradual decrease in error was observed as the valve concentration increased from 1.0 to 4.0 ppg; the AAD dropped from 1.8 to 1.0%.

**Fig. 14:**
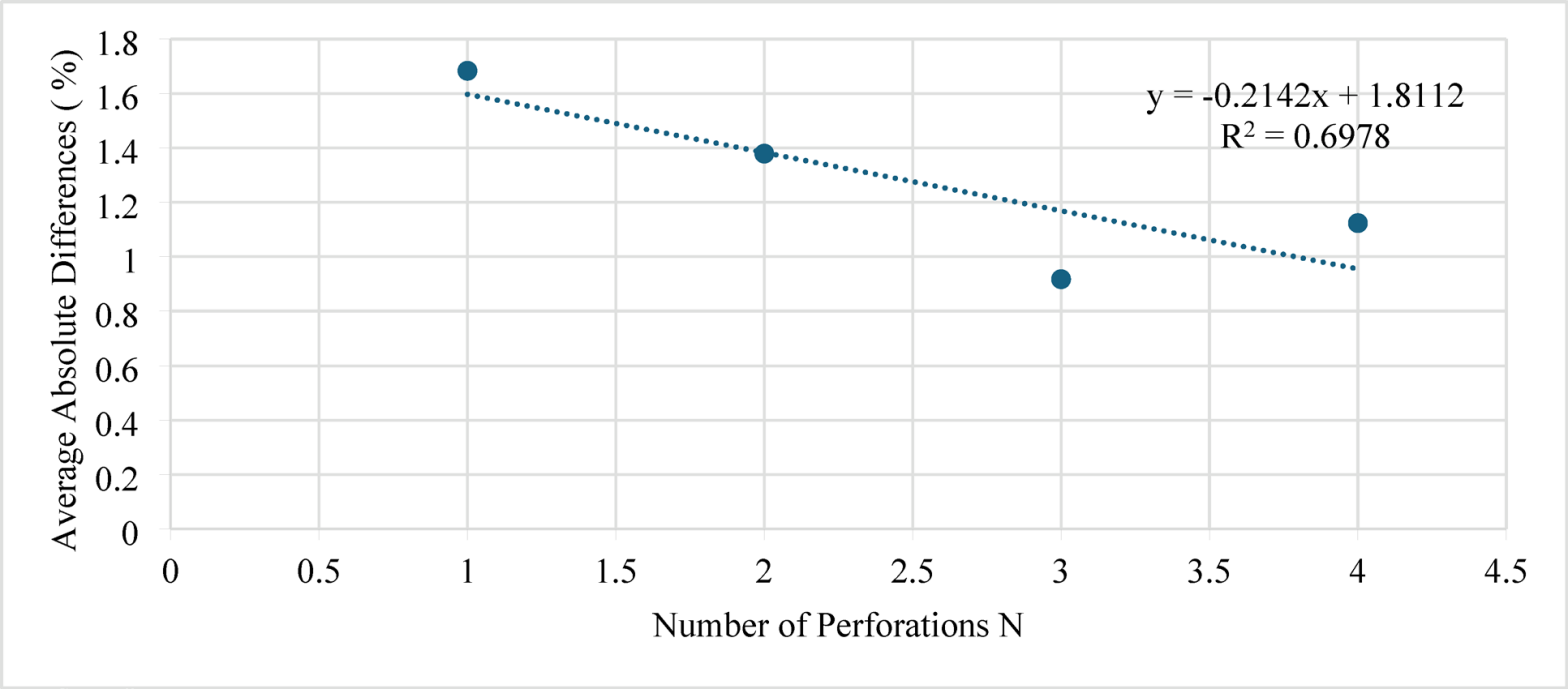
Average absolute difference (AAD) versus number of perforations (N).

The influence of the number of perforations (N) on model performance is presented in Fig. 14. Changing N between 1 and 4 perforations showed a small reduction in the estimation error. For all cases, the absolute difference was less than 2%, demonstrating that the ANN prediction remains stable across a wide range of perforation counts.

Furthermore, sensitivity tests were also performed for the remaining inputs (proppant diameter, injected proppant concentration, Cluster 2 concentration, and perforation angle). These parameters exhibited negligible impact on the average error (changes < 0.5%), confirming that the model’s accuracy is most sensitive to variables directly related to flow distribution and proppant transport.

Overall, the developed ANN model can accurately predict sand-settling behavior over the full range of studied conditions. The sensitivity analysis confirms that injection rate and cluster-specific concentrations are the dominant parameters affecting model accuracy, while geometric and material parameters have a secondary influence.

#### Model B

The effect of each input parameter on the prediction performance of Model B was examined.

**Fig. 15:**
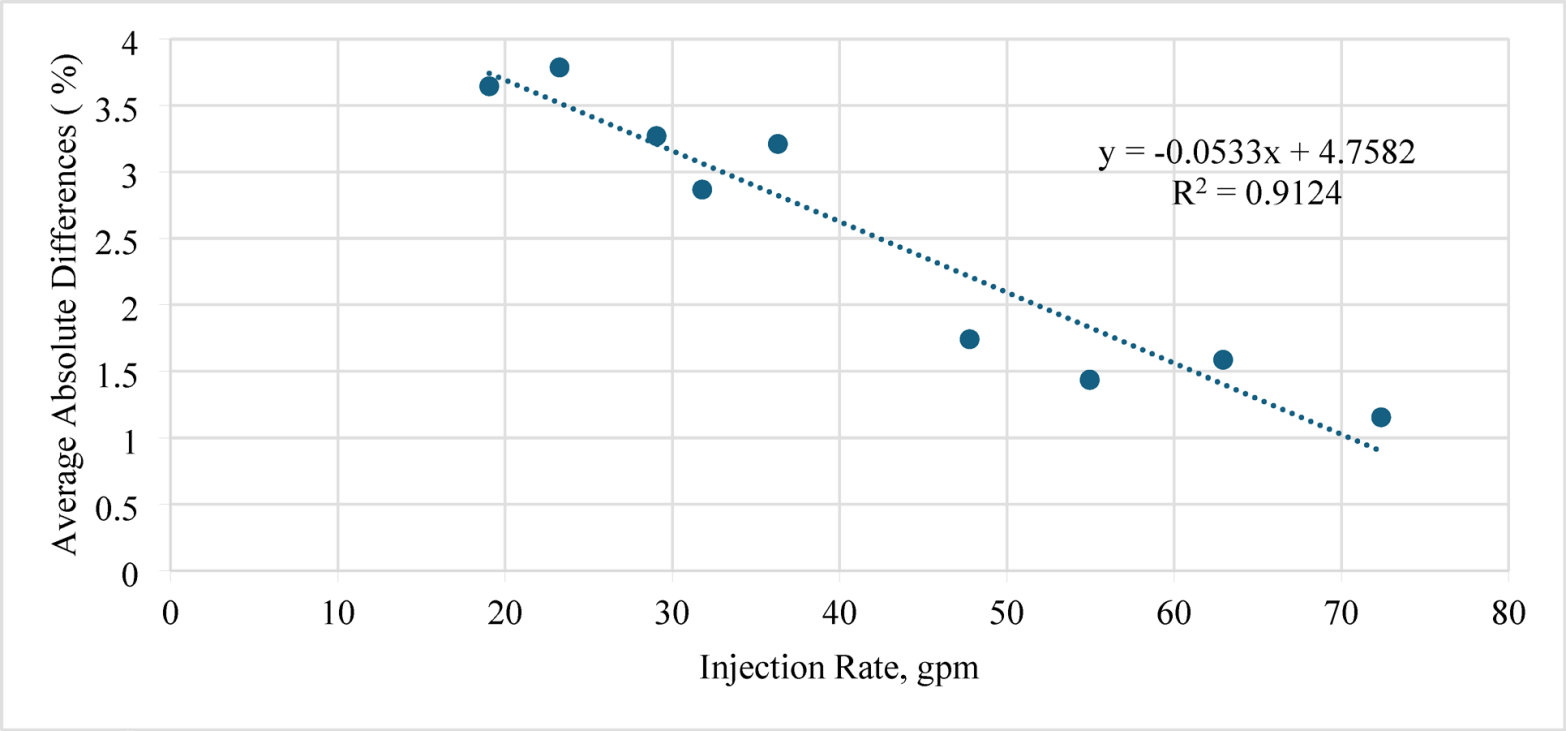
Average absolute difference (AAD) between actual and predicted sand-settling percentage against injection rate.

Figure 15 shows the average absolute difference (AAD) between the actual and predicted sand-settling percentages plotted against the injection rate. Increasing the injection rate from 18 to 73 gpm decreased the AAD from approximately 3.8 to 1.2%, indicating that the ANN model achieves higher accuracy at higher flow rates. Nevertheless, the maximum percentage difference is below 5%, which is acceptable for practical applications.

**Fig. 16:**
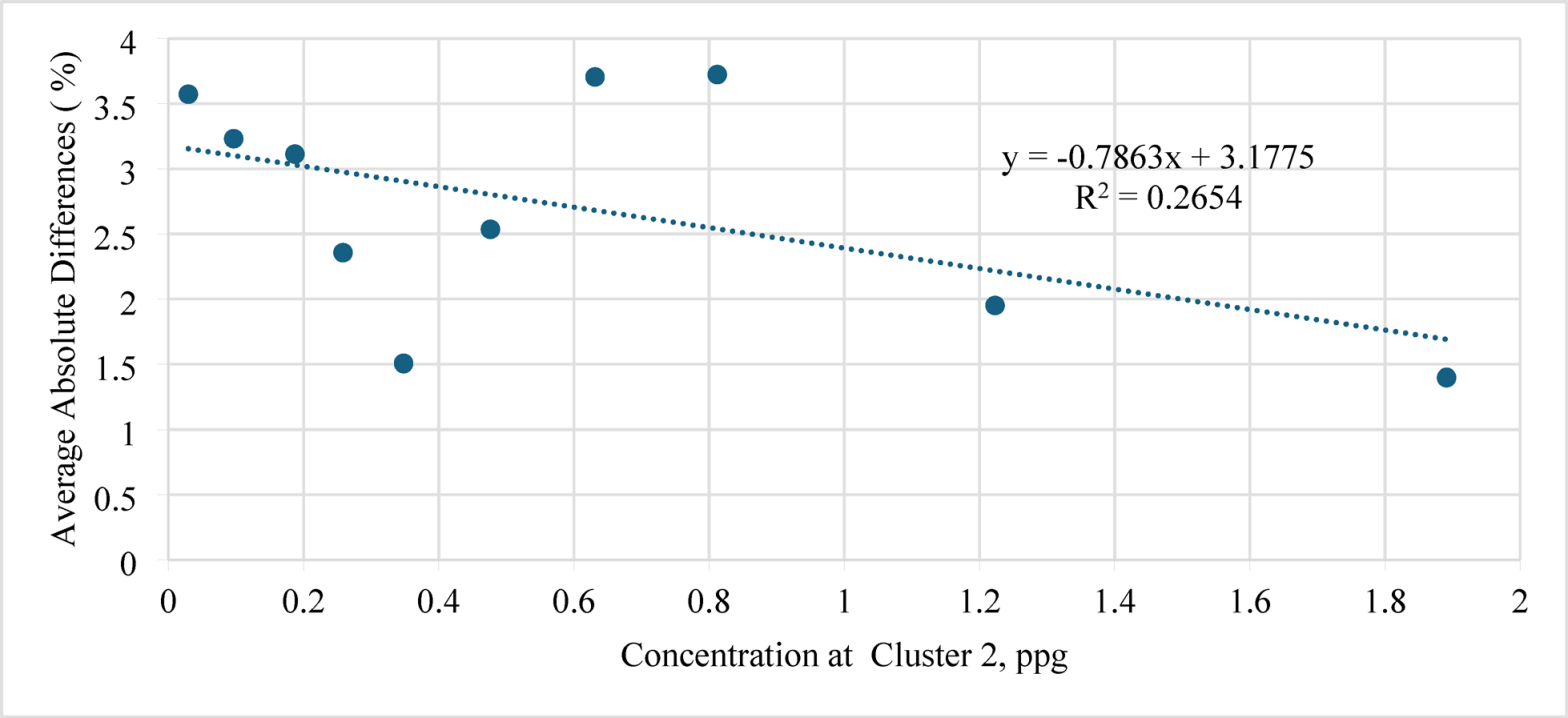
Average absolute difference (AAD) versus concentration at Cluster 2 (ppg).

Moreover, Fig. 16 shows the average absolute difference versus concentration at Cluster 2 (ppg). A gradual reduction in error was observed as Cluster 2 concentration increased from 0.1 to 1.9 ppg; the AAD decreased from 4.0 to 1.5%. This confirms that a balanced proppant distribution in the heel cluster enhances the model’s predictive reliability.

**Fig. 17:**
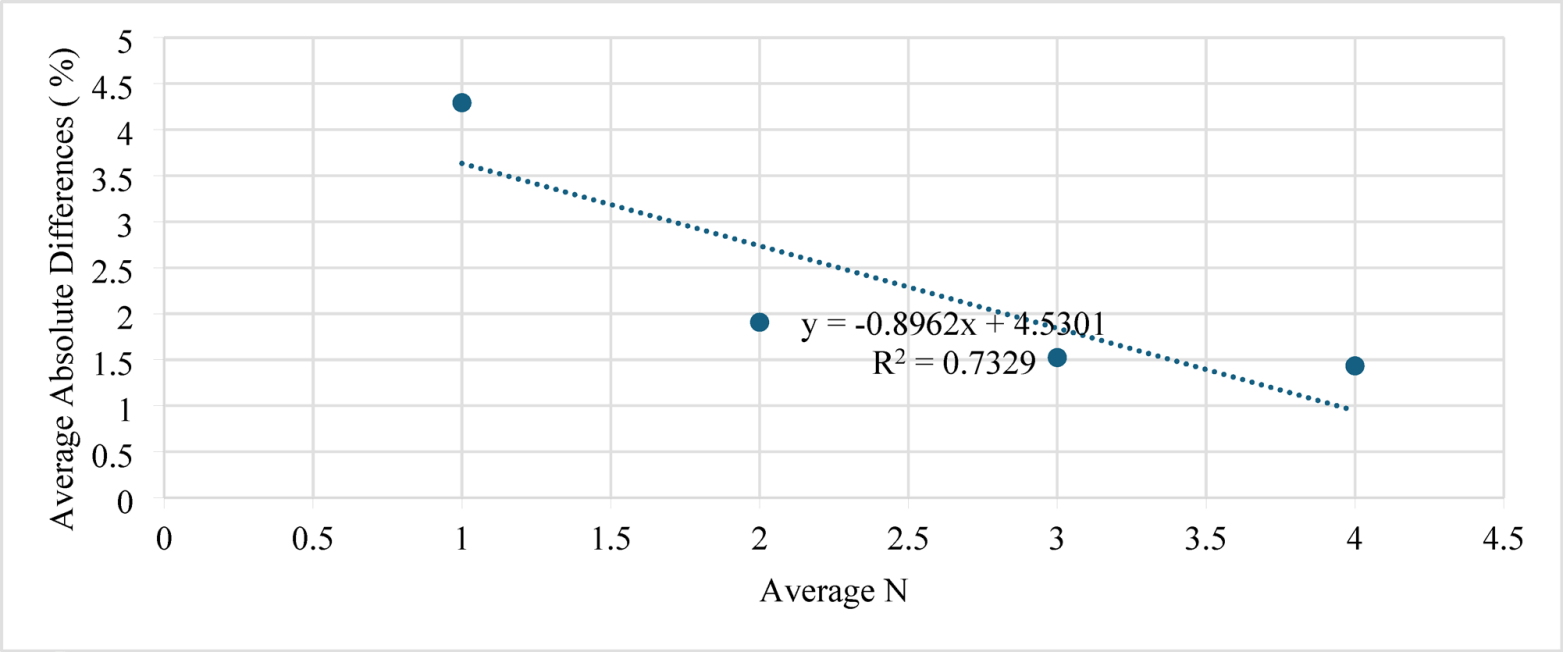
Average absolute difference (AAD) versus number of perforations (N).

The number of perforations (N) also showed a noticeable influence on model performance (Fig. 17): increasing N from 1 to 4 reduced the AAD from 4.25 to 1.5%, confirming that the model remains reliable over a broad perforation range.

The remaining inputs (concentration at cluster 3 and the angle) exhibited negligible variation in AAD (< 0.5%) and, therefore, were not significant contributors to model uncertainty.

Overall, the results show that injection rate and cluster-specific concentrations have the most significant effect on the ANN performance, while geometrical and material properties exert a relatively minor impact. The ANN model can therefore predict sand-settling behavior with high reliability across the studied operating conditions.

### Comparative performance with tree-based models

**Fig. 18:**
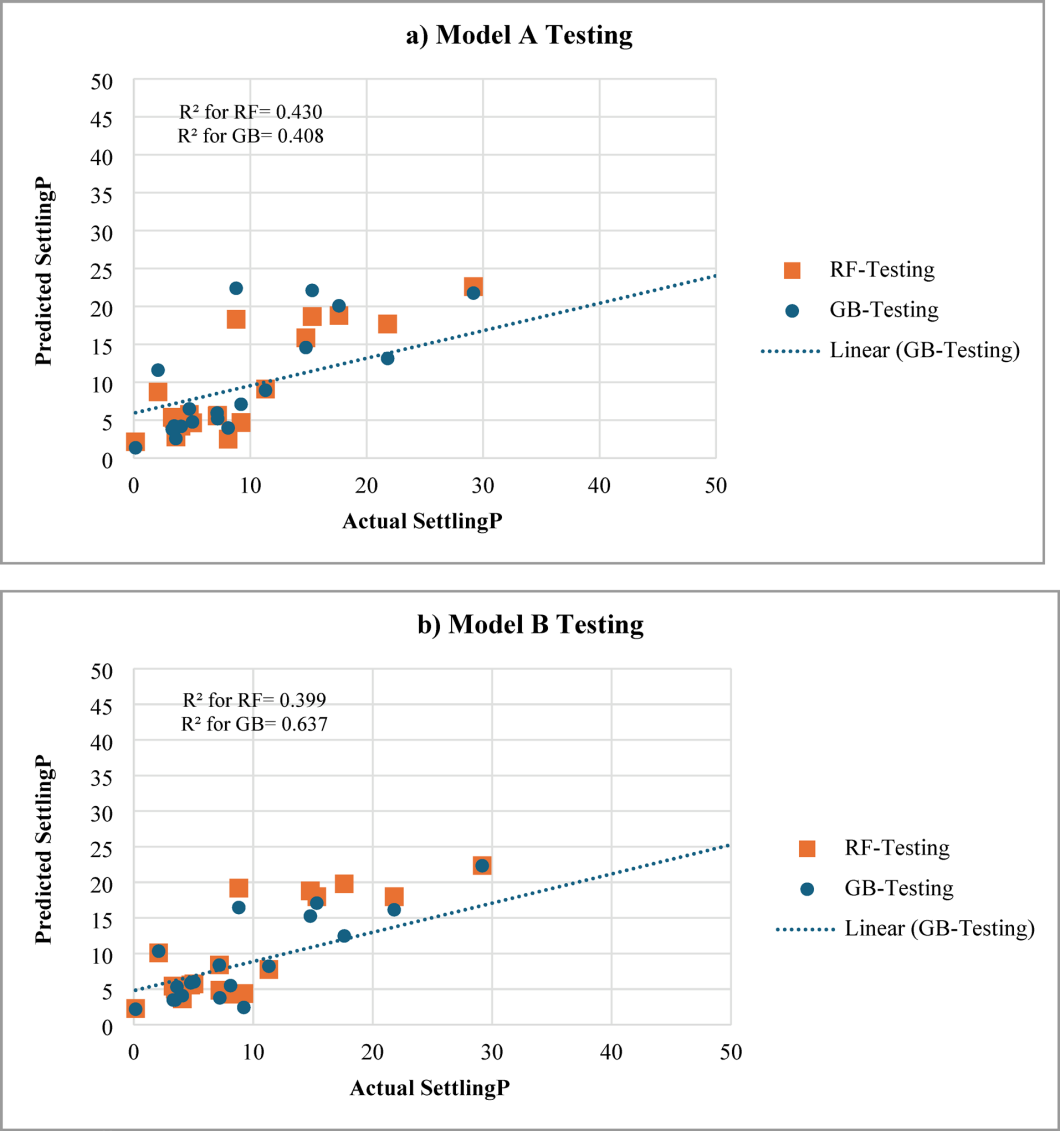
Comparison of predicted versus actual sand settling (%) for random forest (RF) and gradient boosting (GB) models on the testing dataset for (**a**) Model A (9 inputs) and (**b**) Model B (5 inputs). The ANN results are presented in earlier figures for comparison.

To benchmark the ANN performance against other machine-learning algorithms, Random Forest (RF) and Gradient Boosting (GB) regressors were developed using the same datasets and 70/30 training–testing split. The models were optimized using bagging (RF) and boosting (GB) with 800 trees and default hyperparameters in Python. Figure 18 illustrates the predicted versus actual sand-settling percentages for both tree-based methods, and Table 3 summarizes their quantitative performance metrics.

For Model A (9 inputs), the RF and GB models achieved R² values of 0.38 and 0.41 on the testing data, respectively, whereas the ANN achieved an R² of 0.89. Similarly, Model B (5 inputs) yielded R² values of 0.36 (RF) and 0.64 (GB), compared with 0.90 for the ANN. The AAD of the RF and GB models (6%) was significantly higher than that of the ANN (2–3%). These results confirm that ANNs outperform tree-based ensemble models for this regression task, likely due to their superior capability to capture smooth nonlinear interactions among continuous variables in limited-size datasets. This performance advantage reinforces the suitability of ANN models for analyzing and interpreting proppant-settling behavior under varying hydraulic fracturing conditions, as discussed in the following section.

**Table 3:** Comparative performance of artificial neural network (ANN), random forest (RF), and gradient boosting (GB) models for predicting sand-settling percentage in horizontal wellbores under identical 70/30 train–test splits. ANN models consistently achieved higher predictive accuracy and lower error metrics compared with tree-based ensemble methods.

Model	Inputs	Algorithm	*R*² (Testing)	AAD/MAE (%)	RMSE (pp)	MAPE (%)
**A**	9	**ANN**	**0.894**	**1.98**	**2.00**	**8.50**
		RF	0.430	41.34	9.81	112.71
		GB	0.408	44.46	9.82	92.72
**B**	5	**ANN**	**0.899**	**3.02**	**3.20**	**15.80**
		RF	0.399	43.93	9.92	122.10
		GB	0.637	39.84	8.63	114.41

Highlights that both tree-based algorithms achieved moderate predictive capability (R² 0.40–0.64) but with significantly higher average absolute and percentage errors (AAD 40–45%, MAPE > 90%) compared to the ANN models (AAD 2–3%, MAPE < 16%). This indicates that the ANN framework provides a smoother and more reliable mapping of the nonlinear relationships among fracturing parameters and sand-settling behavior.

While Random Forest and Gradient Boosting provided reasonable accuracy (R² 0.36–0.64), the ANN models consistently outperformed both, demonstrating superior capability to capture continuous nonlinear interactions. Nevertheless, these tree-based results confirm that the observed relationships are robust across modeling frameworks and not unique to a specific algorithm.

### Overfitting control, cross-validation, and robustness

Model A is a single-hidden-layer feedforward ANN with nine inputs, ten hidden neurons, and one linear output. Hidden nodes used a tansig activation, and the output layer used a purelin activation. The dataset was randomly permuted using MATLAB’s randperm function with a fixed seed (rand(‘seed’, 200)) to ensure reproducibility, then divided into 70% for training and 30% for testing, where the testing subset remained completely unseen during training. Within the training data, 15% was automatically reserved for validation, allowing MATLAB to apply early stopping once the validation loss ceased to improve, which effectively prevented overfitting.

The network was trained using the Levenberg–Marquardt algorithm (trainlm), and its capacity was deliberately limited to one hidden layer with ten neurons, sufficient to capture nonlinear interactions while minimizing variance. MATLAB’s default input and target normalization further stabilized the training process.

This randomized hold-out validation approach provides an equivalent assessment to cross-validation by exposing the network to randomized subsets and evaluating it on unseen data. In addition, external validation was conducted using the independent dataset of Ahmad and Miskimins^29^, representing slickwater experiments in a 1.5-inch horizontal pipe with different proppant and flow conditions. The strong agreement between predicted and measured sand-settling percentages (R² = 0.816) confirms that the developed ANN models are robust and generalizable to independent datasets.

### Field implementation and practical considerations

While both ANN models provide accurate predictions, their practical applicability differs. Model A, which requires nine input parameters including cluster-specific proppant concentrations, is best suited for controlled laboratory analysis or post-treatment evaluation where detailed flow measurements are available. In contrast, Model B uses only five readily measurable parameters (such as injection rate, number of perforations, and perforation angle) making it more feasible for real-time field deployment. The reduced-input configuration allows field engineers to integrate Model B into digital fracturing dashboards for on-site monitoring and decision support without relying on specialized downhole sensors.

The developed ANN models can be implemented in real-time field operations to estimate sand settling within horizontal wellbores during hydraulic fracturing. In practice, most of the model inputs (such as injection rate, slurry concentration, perforation count, and perforation orientation) are directly available from surface treatment and completion logs. However, some cluster-specific variables (e.g., proppant concentrations at different perforation clusters) are not currently measurable in real time. In such cases, surrogate estimates can be obtained from downhole pressure signatures, distributed acoustic sensing (DAS), or computational flow models that approximate cluster efficiency. Model B, which uses fewer and more accessible inputs, is therefore more suitable for field deployment where data resolution is limited. The ANN model can be embedded in a digital fracturing dashboard to provide continuous estimates of sand settling percentage during pumping operations, enabling engineers to monitor wellbore deposition risk and adjust pumping parameters accordingly. Nevertheless, real-time implementation requires synchronized sensor data and validation under varying field conditions to ensure stability and accuracy of predictions outside laboratory-scale scenarios.

From an engineering perspective, the ANN models confirm and quantify several important trends about sand settling in horizontal wells. Both models identified injection rate as a crucial factor – higher rates strongly reduce settling, which supports the strategy of pumping at high enough rates to minimize sand dropout in laterals. The models also highlighted perforation effects: having more perforations (N) per cluster tends to reduce wellbore sand accumulation, likely because flow is distributed and less sand is left behind. However, in practice, adding too many perforations can reduce the limited-entry effect and lead to unequal cluster efficiency; thus, an optimal balance is needed. The perforation phasing angle being included suggests that oriented perforations (e.g., all on top vs. bottom) might influence sand fallout. For example, if perforations are on the high side of the casing, sand must be lifted upward to exit, possibly increasing settling in the pipe, whereas bottom perforations let gravity assist sand out into fractures – our data and models implicitly capture these effects. Another insight is that proppant size (though not explicitly in Model B) matters: larger sand had higher settling, so choosing smaller proppant or lighter materials could mitigate lateral settling issues. Additionally, the cluster location (as represented by differences between cluster 1, 2, 3 outputs in Model A) plays a role – generally, we observed that the first cluster (nearest the heel) often received more sand than the last cluster (toe) for certain configurations, a phenomenon often reported as uneven cluster distribution in field fracturing (Crespo et al. 2013; Ahmad and Miskimins 2020). Our ANN, by including cluster output variables, indirectly captured this effect: for instance, if cluster 3 (toe-most) concentration is low, it tends to correlate with higher overall settling (because sand didn’t reach that far and fell out earlier).

Comparing these ANN models to traditional empirical models, it is evident that the ANN offers improved accuracy and flexibility. The best published empirical correlations for similar scenarios Ahmad and Miskimins; and Alajmei^15,29^ achieved multiple correlation coefficients around 0.85–0.90 in their validation, whereas our Model A reaches R² 0.96 on the complete dataset and 0.89 on truly unseen data. While not a direct one-to-one comparison (since the datasets differ), this suggests the ANN can match or exceed the predictive power of empirical formulas. More importantly, the ANN does so without requiring the user to compute dimensionless groups or apply specific correlation equations – it takes raw input parameters and directly yields an output. This can simplify integration into field operations: for example, a real-time system could feed in measured pump rate, slurry concentration, etc., into the ANN and get a continuous estimate of sand settling or proppant distribution. Furthermore, the ANN can be retrained or updated when new data becomes available, gradually improving its accuracy or expanding its range of applicability (whereas empirical correlations are static once formulated).

Regarding the limitations, the current ANN models are data-driven and thus rely on the quality and representativeness of the experimental data. If field conditions fall outside the range of our tests (e.g., much higher viscosity fluids, different pipe sizes, presence of gas, etc.), the model may need retraining or might not directly apply. Also, the ANN doesn’t inherently provide a physical explanation; it is essentially a black box. However, we can extract some interpretability by analyzing variable importance (e.g., via sensitivity analysis: perturbing one input and seeing the effect on the output).

### Model validation

**Fig. 19:**
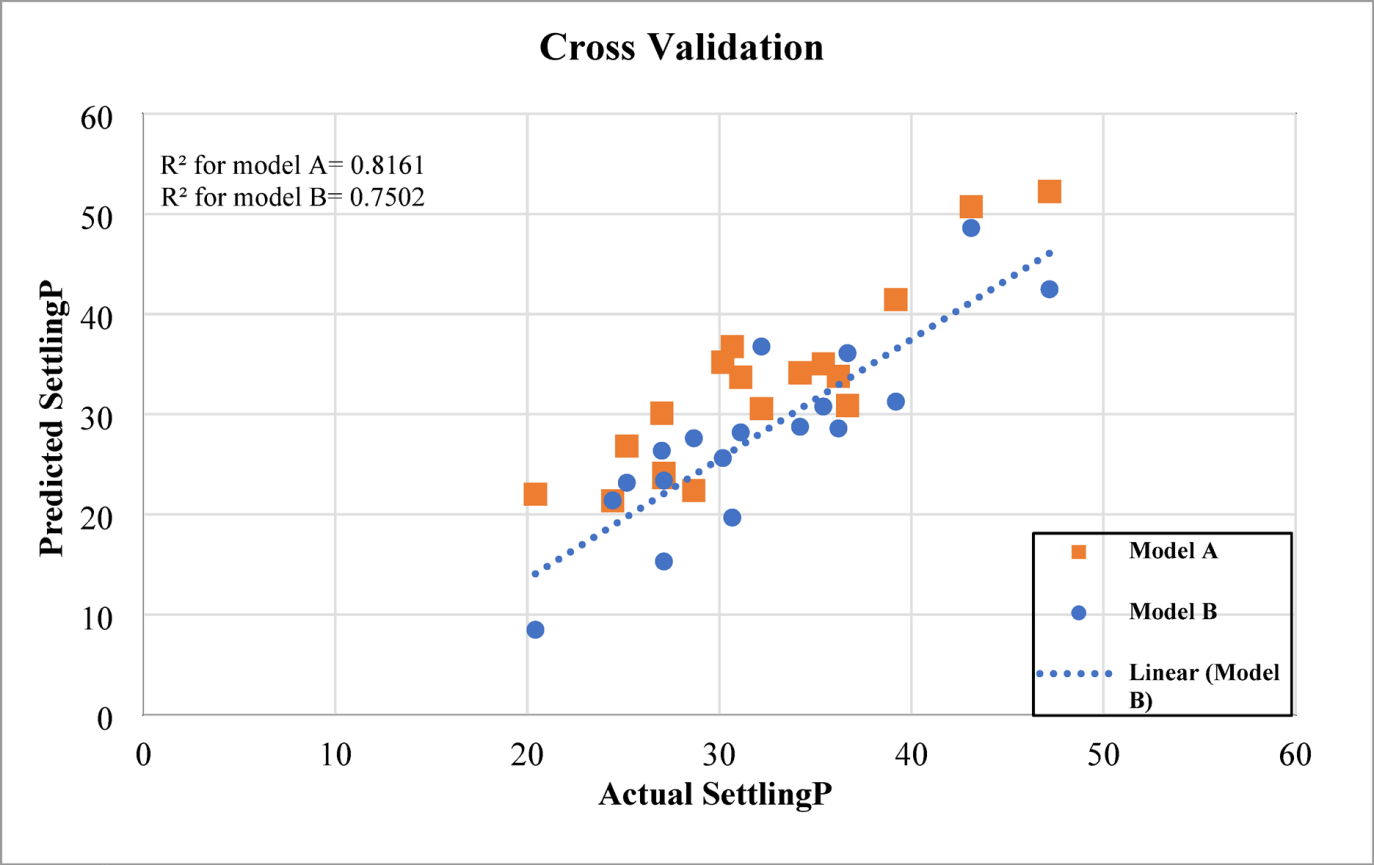
Cross-validation results of the ANN models using external data from ahmad and miskimins^29^.

The developed ANN models were further validated using an independent dataset obtained from Ahmad and Miskimins^29^, which reports proppant transport behavior in horizontal wellbores under low-viscosity fluid conditions. The validation results, shown in Fig. 19, indicated that Model A achieved an R² of 0.8161, while Model B achieved an R² of 0.7502. The slightly lower performance compared to the training phase is expected, as the settling percentages in the validation dataset lie in the upper range of the training data, where prediction errors typically increase. In addition, one of the input parameters, the proppant diameter, was outside the range used in training, though its relatively low correlation coefficient (CC) minimized its effect on model degradation. As anticipated, Model A slightly outperformed Model B due to the inclusion of more input variables, whereas Model B demonstrated acceptable accuracy with fewer inputs. It is also noted that the validation dataset did not report proppant concentration at the outlet valve, which limited the evaluation of that parameter. Overall, both models showed reasonable predictive capability when tested on external data, confirming their robustness and generalization potential.

**Fig. 20:**
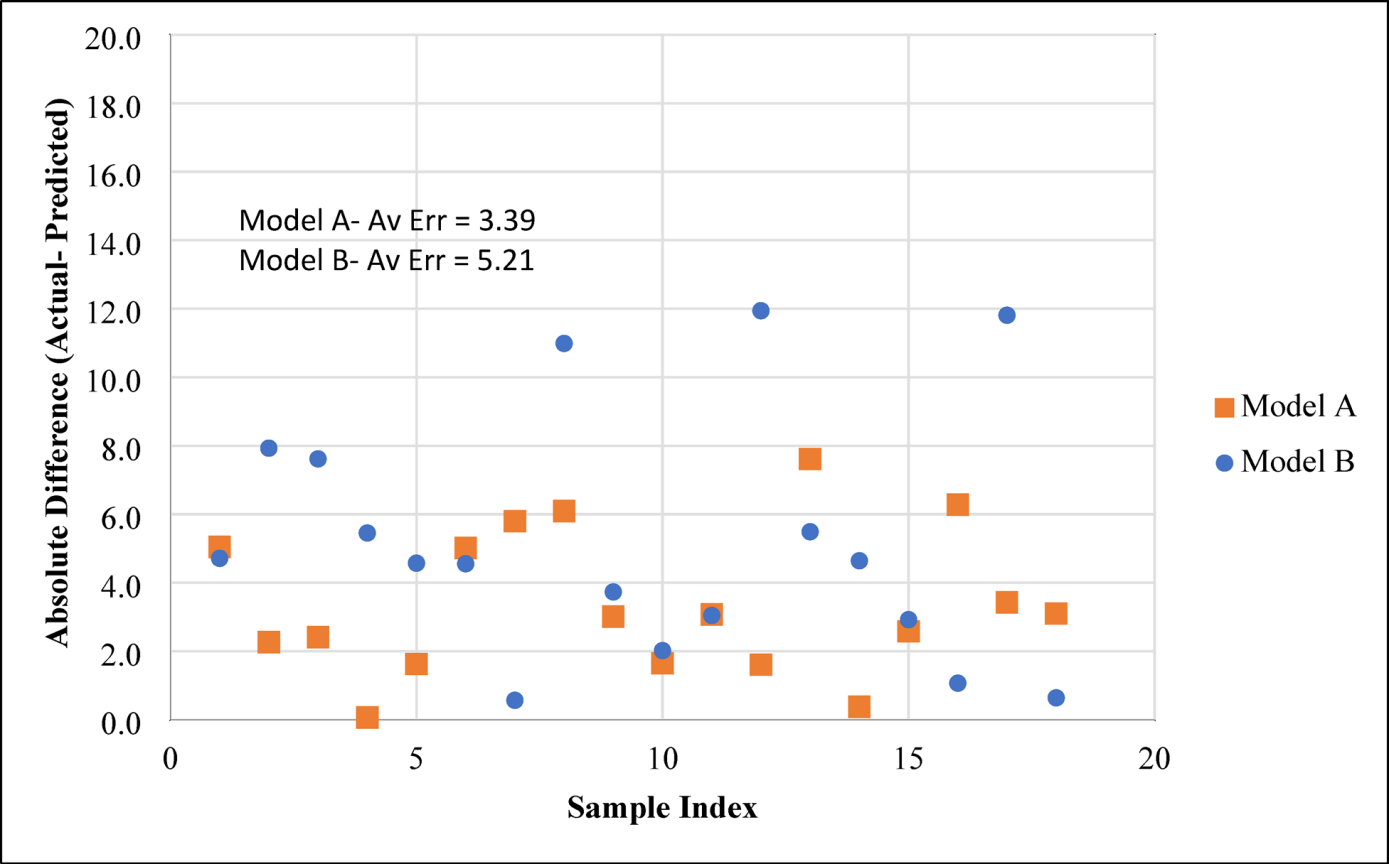
Error distribution of the ANN models during model validation.

The error distribution of the cross-validation results is shown in Fig. 20. Model A exhibited an average absolute error of 3.39, while Model B recorded a higher error of 5.21. The spread of errors indicates that Model A generally produced more consistent predictions across the dataset, with fewer large deviations compared to Model B. This improved performance is attributed to the inclusion of additional input variables in Model A, which provided the network with more information to capture the nonlinear interactions governing sand settling. In contrast, Model B, while simpler, showed slightly higher variability in prediction accuracy due to its reduced input set. Nevertheless, both models maintained acceptable error levels, confirming their capability to generalize when applied to external datasets.

These findings confirm that both ANN models are robust within the conditions tested. However, their applicability to other hydraulic fracturing environments should be considered within the limitations of the present study.

The external validation conducted in this study utilized the dataset reported by Ahmad and Miskimins^29^, which corresponds to low-viscosity slickwater experiments in a 1.5-inch-diameter horizontal pipe with 100-mesh and 40/70 sand proppants. While this provides an independent test confirming the model’s ability to generalize to unseen data under comparable conditions, it does not encompass other fluid types or geometries. Consequently, the current validation is limited to fresh-water (slickwater) systems and a single pipe diameter. Broader validation across fluids with higher viscosity (e.g., polymer gels, foams), different pipe sizes, or field-scale datasets is proposed as future work to further assess model transferability and robustness under varied hydraulic-fracturing scenarios.

### New correlation

To enable straightforward engineering use of the developed ANN models, the trained networks were expressed as explicit mathematical correlations. This approach allows practitioners to calculate sand settling percentages directly without requiring machine learning code. The correlation follows the forward-pass formulation of a multilayer feedforward neural network; it uses the weights and biases of the trained ANN models, listed in Table 4 and Table 5.

This neural network-based correlation is represented by two equations, Eq. (7) and Eq. (8), both of which enable accurate determination of the sand settling percentage in horizontal wellbore.

7 $$\:Sp=\left[{\sum\:}_{i=1}^{N}{w}_{2i}\hspace{0.17em}tansig\left({\sum\:}_{j=1}^{J}{w}_{1ij}{x}_{j}+{b}_{1j}\right)\right]+{b}_{2}$$

8 $$\:Sp=\left[{\sum\:}_{i=1}^{N}{w}_{2i}\left(\frac{2}{1+{e}^{-2\left({\sum\:}_{j=1}^{J}{w}_{1ij}{x}_{j}+{b}_{1j}\right)}}\right)\right]+{b}_{2}$$

Where Sp is the predicted sand settling percentage (%), N is the number of hidden neurons, and J is the number of input parameters (nine for Model A and five for Model B). The terms Xj are the normalized input variables, W1ij and b1j are the input-to-hidden weights and biases, and W2i and b2 are the hidden-to-output weights and biases. The hidden neurons use the hyperbolic tangent transfer function (tansig), defined as $\:\text{t}\text{a}\text{n}\text{h}\left(u\right)=\frac{2}{1+{e}^{-2u}}-1$. The values of the weights and biases needed for Eq. (7) and Eq. (8) are listed in Table 4 and Table 5., making the process easier and more understandable for users.

**Table 4:** The values of the weights and biases for determining the sand settling percentage in a horizontal wellbore, using model A.

Number of Neurons	Input layer										Output Layer	
	Weights (w1)									Biases (b1)	Weights (w2)	Bias (b2)
	X1	X2	X3	X4	X5	X6	X7	X8	X9			
1	-1.039	-1.859	1.195	1.145	-1.773	0.919	0.381	0.305	-1.899	0.886	-0.165	2.090
2	2.324	-0.865	1.868	1.799	0.586	-0.848	1.299	1.128	-1.595	-1.566	1.761	
3	0.814	1.403	0.634	0.438	1.514	-1.634	0.247	0.612	0.827	0.829	-0.070	
4	-0.736	-1.691	-1.204	-1.738	1.736	-0.635	0.862	-0.590	-0.128	-0.530	-0.684	
5	0.472	0.044	-1.498	-1.225	1.811	-0.482	-0.516	0.261	-0.484	1.679	-0.144	
6	0.513	-0.259	-1.005	1.479	0.850	-0.493	-0.430	-1.015	-0.716	-0.908	1.405	
7	-1.431	0.005	-0.600	0.361	0.921	-0.713	-1.053	0.546	1.047	-0.818	0.089	
8	1.062	-1.663	1.562	-1.524	-0.730	-0.421	0.017	-1.404	-1.878	-0.858	0.821	
9	2.249	-1.991	-3.893	-0.058	-0.971	0.141	-0.702	-0.536	0.451	-0.396	-0.646	
10	-2.993	0.306	0.890	1.124	-1.624	0.705	-1.993	0.041	1.683	-1.174	0.967	

**Table 5:** The values of the weights and biases for determining the sand settling percentage in a horizontal wellbore, using model B.

Number of Neurons	Input layer						Output Layer	
	Weights (w1)					Biases (b1)	Weights (w2)	Bias (b2)
	X1	X2	X3	X4	X5			
1	-2.235	-0.355	-0.271	-0.459	-0.096	-1.129	1.020	-0.198
2	-3.041	-1.065	-5.693	1.754	5.226	-3.888	-1.678	
3	-2.303	2.177	3.052	0.883	1.755	2.467	0.790	
4	-2.276	-2.260	0.253	1.211	-1.908	0.622	-1.848	
5	-2.649	1.155	-3.584	3.697	-1.641	1.094	2.740	
6	6.046	-1.695	-1.456	-4.224	-0.357	0.333	0.613	
7	1.421	0.701	-0.817	-1.428	-0.763	-0.050	-0.637	
8	1.146	-1.088	-4.049	4.276	1.552	0.877	2.706	

To illustrate reproducibility and provide a practical guide for using the developed correlations, a worked example is presented here demonstrating how the published ANN weights and biases (Tables 3 and 4) can be used to reproduce one of the model predictions.

For Model A, the normalized input parameters for a representative experimental condition are: X=[0.21, 0.47, 0.25, 0.33, 0.28, 0.26, 0.30, 0.50, 0.42].

These correspond respectively to the normalized values of:

proppant diameter, actual injection rate, injected proppant concentration, Cluster 1 concentration, Cluster 2 concentration, Cluster 3 concentration, valve concentration, number of perforations (N), and perforation angle.

The original (un-normalized) experimental input values that produce this normalized vector are summarized in Table 6. Normalization was performed using MATLAB’s mapminmax function, which linearly scales each parameter to the 0–1 range based on the minimum and maximum values observed in the training dataset.

**Table 6:** Example of original (un-normalized) and normalized input values used for model A prediction.

Parameter	Original Value	Units	Normalized Value
Proppant diameter	0.00025	in	0.21
Injection rate	42.0	gpm	0.47
Injected concentration	0.90	ppg	0.25
Cluster 1 (ppg)	0.80	ppg	0.33
Cluster 2 (ppg)	0.59	ppg	0.28
Cluster 3 (ppg)	0.55	ppg	0.26
Valve (ppg)	0.46	ppg	0.30
Number of perforations (N)	2	–	0.50
Perforation angle	90	°	0.42

The ANN forward-propagation is computed using Eq. 7 and the weights and biases provided in Table 4. Substituting the above normalized input vector and the published parameters yields a predicted sand-settling percentage of 9.4%, compared with the experimentally measured value of 9.3%. The small deviation (< 1%) confirms that the published ANN weights and biases reproduce the reported model predictions exactly.

This example demonstrates how the ANN equations can be implemented by practitioners using the published coefficients, ensuring model transparency and reproducibility.

### Limitations and scope of the study

The applicability of the developed ANN models should be interpreted within the scope of the experimental conditions under which they were trained. The laboratory dataset was generated using fresh water (slickwater) as the base fluid, a 1.5-inch inner-diameter transparent pipe, and two types of quartz proppant (100-mesh and 40/70-mesh sands). These conditions were intentionally selected to represent a controlled and repeatable configuration that emphasizes the fundamental mechanisms of proppant settling under low-viscosity, high-settling environments, considered the most challenging scenario for suspension. Using slickwater ensures that the observed settling trends are conservative, while the fixed pipe size enables direct comparison among runs without geometric scaling uncertainty. The use of two well-characterized sand types allowed isolation of size effects without introducing compositional or density variability.

Accordingly, the developed ANN models are best suited for predicting sand settling behavior in water-based fracturing systems and laboratory-scale or field configurations of similar diameter ranges. Extrapolation to higher-viscosity fluids (e.g., polymer gels, foams), larger wellbore diameters, or alternative proppant materials (e.g., lightweight ceramics or resin-coated sands) should be approached with caution, as these conditions introduce additional fluid–particle and scale-dependent effects not captured in the present dataset. Extending the framework to such systems would require retraining or recalibrating the ANN with new experimental data encompassing those ranges. Future work will therefore focus on expanding the dataset to include different fluid rheologies, geometries, and proppant types to broaden the model’s generalizability.

In addition, external validation in this study was performed using data from a single independent source^29^ under slickwater conditions and a fixed pipe geometry. Future work should include validation on other fluid systems and larger-scale geometries to extend the model’s range of applicability.

## Conclusions

This study aims to develop a quick and accurate model for predicting the settling of sand proppants in a horizontal wellbore after hydraulic fracturing using the artificial neural network (ANN) technique. Based on this study, the following conclusions can be stated:

- The study included 9 input parameters, including: The proppant diameter, actual injection rate, injected proppant concentration, the exited proppant concentrations from different clusters, angle, and the number of perforations, and the output is the sand settling percentage in the horizontal wellbore.
- Two models were built: Model A, which predicts the sand settling percentage using all parameters (9 inputs), and Model B, where the number of inputs was reduced to 5. Correlation coefficient analysis was utilized to identify the strength of the relationships between input variables and the output variable, facilitating the reduction of input variables.
- The work incorporated benchmarking with Random Forest and Gradient Boosting algorithms, confirming that the ANN models achieved far superior predictive accuracy. The trained ANN weights and biases were also published as explicit equations, allowing straightforward engineering use without specialized software. These enhancements strengthen the study’s practical value and support future applications of data-driven models for optimizing hydraulic fracturing designs.
- Cross-validation using external experimental data confirmed the robustness of both models, with Model A achieving an R² of 0.816 and Model B achieving an R² of 0.750. Model A consistently outperformed Model B due to its larger input set, while Model B retained acceptable accuracy despite fewer variables. These results highlight the models’ ability to generalize to independent datasets and demonstrate their potential applicability for real-world proppant settling prediction.
- Overall, Model A showed better predictions for the sand settlement compared to Model B. The average absolute differences for the testing data are 1.98 and 3.02 for Model A and Model B, respectively. The R2 values are 0.894 and 0.89 for Model A and Model B, respectively.

## Data Availability

The datasets used and/or analysed during the current study available from the corresponding author on reasonable request.

## Abbreviations

ANN: Artificial Neural Network (-)
AAD: Average Absolute Difference (%)
MSE: Mean Squared Error(%)
MAPE: Mean Absolute Percentage Error(%)
R²: Coefficient of Determination(-)
Gpm: Gallons per minute(Gal/min)
Ppg: Pounds per gallon(Ib/gal)
SPF: Shots per foot(Shots/ft)
in: Inches(In)
kg/m³: Kilograms per cubic meter(Kg/m3)
N: Number of perforations(-)
θ: Angle(degrees (°))
ρ: Density(Kg/m³)
µ: Fluid viscosity(cP)
d: Proppant diameter(In)
Q: Actual injection rate(Gal/min)
C: Injected proppant concentration(Kg/m³)
Cluster 1, 2, 3: Proppant concentration in clusters 1, 2, and 3(Ib/gal)
Valve: Valve pressure(Ib/gal)
Settling P: Sand settling percentage(%)
